# Vanadate Retention by Iron and Manganese Oxides

**DOI:** 10.1021/acsearthspacechem.2c00116

**Published:** 2022-08-05

**Authors:** Macon J. Abernathy, Michael V. Schaefer, Roxana Ramirez, Abdi Garniwan, Ilkeun Lee, Francisco Zaera, Matthew L. Polizzotto, Samantha C. Ying

**Affiliations:** † Stanford Synchrotron Radiation Lightsource, SLAC National Accelerator Laboratory, Menlo Park, California 94025, United States; ‡ Department of Earth and Environmental Science, New Mexico Institute of Mining and Technology, Socorro, New Mexico 87801, United States; § Environmental Sciences Department, University of California-Riverside, Riverside, California 92521, United States; ∥ Department of Chemistry, University of California-Riverside, Riverside, California 92521, United States; ⊥ Department of Earth Sciences, University of Oregon, Eugene, Oregon 97403, United States; ¶ Environmental Toxicology Graduate Program, University of California-Riverside, Riverside, California 92521, United States

**Keywords:** adsorption, surface complexation, redox, goethite, birnessite

## Abstract

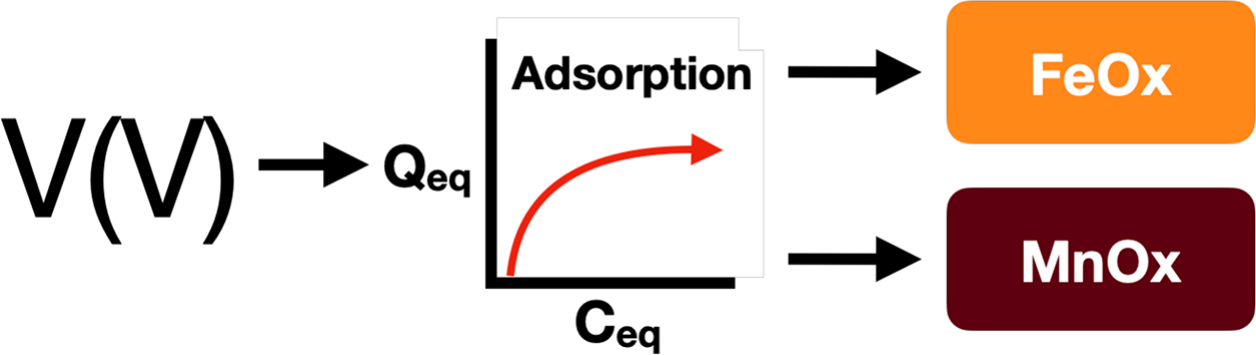

Anthropogenic emissions of vanadium (V) into terrestrial and aquatic surface systems now match those of geogenic processes, and yet, the geochemistry of vanadium is poorly described in comparison to other comparable contaminants like arsenic. In oxic systems, V is present as an oxyanion with a +5 formal charge on the V center, typically described as H_*x*_VO_4_^(3–*x*)–^, but also here as V(V). Iron (Fe) and manganese (Mn) (oxy)hydroxides represent key mineral phases in the cycling of V(V) at the solid–solution interface, and yet, fundamental descriptions of these surface-processes are not available. Here, we utilize extended X-ray absorption fine structure (EXAFS) and thermodynamic calculations to compare the surface complexation of V(V) by the common Fe and Mn mineral phases ferrihydrite, hematite, goethite, birnessite, and pyrolusite at pH 7. Inner-sphere V(V) complexes were detected on all phases, with mononuclear V(V) species dominating the adsorbed species distribution. Our results demonstrate that V(V) adsorption is exergonic for a variety of surfaces with differing amounts of terminal −OH groups and metal–O bond saturations, implicating the conjunctive role of varied mineral surfaces in controlling the mobility and fate of V(V) in terrestrial and aquatic systems.

## I Introduction

Geogenic and anthropogenic emission of vanadium (V) into the biosphere poses an increasing threat to water quality,^1−3^ human health,^4^ and sensitive ecological systems.^5,6^ Vanadium has demonstrable toxicity at exposures as low as 1.2–80 μg/L for sensitive aquatic species,^5−7^ and elevated concentrations have been shown to alter microbial community structure^8,9^ and reduce crop yields.^10,11^ Increases in steel demand and the extraction and combustion of fossil fuels have drastically increased the mobilization of V from the Earth’s crust over the past century.^7,12−14^ Recent estimates suggest that anthropogenic emissions of V into the biosphere now exceed V emissions from geologic processes.^13^ However, with few exceptions, the source of mobile V in subsurface environments appears to be dominated by weathering processes.^7,14^ This is most relevant to regions with aquifers developing on parent material rich in Fe^III^ and Al^III^ (hydr)oxides due to the high weight percentages of V^III^ and V^IV^ substitution that can occur in these minerals.^15−22^ Weathering processes drive the release of V from these mineral phases into the groundwater-sediment matrix leading to elevated pore water concentrations, as well as remove V through adsorption processes.^19,23−25^ Such sediment weathering has resulted in V mobilization to aquifer pore spaces resulting in well water contamination throughout California.^1,2^

Vanadium is a redox active metal present in the +3, +4, and +5 oxidation states in terrestrial environments.^2,7^ The solubility, and thus mobility, of V is highly dependent on its oxidation state, with solubility increasing with oxidation state at circumneutral pH. Additionally, V mobility in soils is greater in the absence of organic matter.^7,26,27^ Vanadium(V) species are the most mobile forms of V in terrestrial and aquatic environments, and their high degree of toxicity makes them a particular concern for human health.^5,26−28^ Typically, V^V^ is observed as a vanadic acid derivative (H_*n*_VO_4_^*n*–3^) at environmentally relevant concentrations. However, even at concentrations as low as 50 μM, a small percentage of the total vanadate polymerizes to form polyvanadate species that have unique biological and geochemical behaviors.^29−31^ Accordingly, V^V^ in this text will refer to total vanadate concentrations as a way to describe this distribution, which is dominated by H_2_VO_4_^–^ at circumneutral pH.

The fate of vanadate in the environment is largely controlled by surface processes.^32^ Early work by Wehrli and Stumm^32,33^ considered the effects of adsorption on V^IV^ retention and oxidation, and subsequent work by Peacock and Sherman^34^ examined the effects of vanadate complexation by the Fe^III^-hydroxide goethite. More recent studies have primarily focused on V retention by whole soils,^19,25,35−38^ or individual minerals.^31,39,40^ In all cases, surface interactions with the soil phases result in the removal of vanadate from the aqueous phase, decreasing its availability for uptake.^36,37,41^ Although few spectroscopic studies have examined the mechanism of vanadate retention by mineral phases in detail, V has been shown to form covalent, inner-sphere complexes with a host of mineral phases.^31,39−41^ In the case of Fe^II^-bearing Fe oxides, inner-sphere complexation with vanadate can result in electron transfer from structural Fe^II^ resulting in adsorbed or incorporated V^IV^.^40^ However, many details related to these surface-mediated retention processes are unknown. As such, more research has been called for by both scientists^3,7^ and regulators^42,43^ to further our understanding of the geochemical controls that govern the mobility of V in the subsurface with the goal of improving the management and reclamation of sites impacted by V contamination.

The goal of this study is to examine the mechanisms of V^V^ retention by selected Fe and Mn (hydr)oxide phases that control the fate and transport of other geogenic contaminants.^34,39,44−49^ Thermodynamic parameters derived from Langmuir theory are used to assess the role of sorbent crystallinity and surface area on V^V^ complexation by manganese and iron oxides. The dominant modes of surface complexation with increasing V^V^ concentration are assessed spectroscopically to corroborate the adsorption affinities observed under equilibrium conditions.

## II Materials and Methods

### Mineral Acquisition and Synthesis

Pyrolusite (Pyr) was purchased as ≥99% MnO_2_ from Sigma-Aldrich, and goethite (Gt) was purchased from Strem Chemicals. Hematite (Hm), two-line ferrihydrite (Fhy), and hexagonal K-birnessite (Birn) were synthesized following the protocols of Cornell and Schwertmann^50^ and McKenzie,^51^ respectively. All oxides were finely ground with an agate mortar and pestle prior to characterization and use. Mineral synthesis is summarized in Section VII of the Supporting Information.

### Mineral Characterization

All minerals were characterized by powder X-ray diffraction (XRD) using a Siemens D500 diffractometer equipped with a Cu Kα X-ray source operating at 40 kV. Randomly oriented powders were mounted in an aluminum sample holder, and data were collected between 2 and 80° 2θ and 0.01° step size. Alignment of the diffractometer was previously calibrated using a quartz standard. JADE software (Materials Data, Inc.) was used for background subtraction, and peak positions and intensities were matched against reference data from the Joint Committee on Diffraction Standards Mineral Database as well as the American Mineralogist Crystal Structure Database.

Surface area and pore-size analysis was performed using a Quantachrome Nova 2000e analyzer. Surface area analysis and collection of the corresponding pore-size distribution was conducted at 77.35 K using multipoint BET and adsorption–desorption Barrett–Joyner–Halenda (BJH) methods. All characterization data is presented in Section I of the Supporting Information.

### Sorption Experiments

Ten concentrations of Na_3_VO_4_ were prepared, ranging from 5 to 2000 μM, and a non V control. Treatment solutions were composed of ultrapure water buffered with 10 mM PIPES that was brought to a pH of 7.00 using less than 400 μL of 12 M NaOH per liter of solution. Ionic strength was adjusted with the addition of NaCl to a final concentration of 25 mM. The sorption experiments were carried out in static batch reactors using 50 mL vials with oxide loadings of 1 g L^–1^ for birnessite, goethite, and ferrihydrite and 100 mg L^–1^ for hematite and 2 g L^–1^ for pyrolusite due to their high and low surface areas, respectively.

All sorption experiments were performed in triplicate, and vials were stored in the dark with daily manual shaking. The sorption experiments were allowed to equilibrate for at least 3 weeks before syringe-filtration through a 0.22 μm PES membrane. All solutions were analyzed for dissolved V, Mn, and Fe using inductively coupled plasma-optical emission spectrophotometry (ICP-OES). Solid phase samples were harvested for analysis by X-ray adsorption spectroscopy (XAS) *via* filter deposition onto 0.45 and 0.22 μm MCE membranes.

Plots of adsorbed V^V^ (*q*_eq_) as a function of the equilibrium concentration (*C*_eq_) were evaluated for the suitability of a single or two-site Langmuir model by examining the isotherms after applying Scatchard transformations ( vs *q*_eq_, eq 2).^52,53^ All data were found to exhibit two-site characteristics^52^ and were modeled using the two-site Langmuir (2L) model described by eq 1.^54^
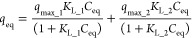
1where: *q*_eq_ is the amount of V^V^ adsorbed to the oxide surface at equilibrium in μmol g^–1^

*C*_eq_ is the aqueous equilibrium concentration of V^V^ (μM)

*q*_max_ is the adsorption capacity of a given site (μmol g^–1^)

*K*_L_ is the Langmuir constant of a given site (L mol)

The two-site Langmuir model (2L model) was selected to model the adsorption interaction on the basis of the multilinearity of the corresponding Scatchard plots and linearized single-site isotherm (Figures S2 and S3, eq 2) for each V–oxide interaction (Supporting Information Section II),^52,55,56^
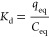
2where *q*_eq_ is in units of μmol g^–1^ and *C*_eq_ is in μM. This model is easy to implement and interpret, while yielding parameters that are amenable to energy calculations and reactive transport modeling.^57−60^ A generalized reduced-gradient nonlinear least-squares fitting algorithm^61^ was used to fit the adsorption models to each data set using a global optimization method.^62^ Optimization was performed by minimizing the weighted sum of squared residuals. Further details on calculations can be found in the Supporting Information. No unexpected safety hazards were encountered in the course of the experiments or analysis.

### Calculation of Thermodynamic Parameters

Once *K*_L_ values were obtained from the 2L models, the equilibrium Gibbs free energy of adsorption (Δ*G*°_ads_) was estimated for V^V^ at each site using the eq 3 developed by Liu (2009):^63,65^
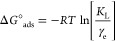
3where *R* is the gas constant (8.314 J K^–1^ mol^–1^), *T* is the absolute temperature in kelvin, *K*_L_ is the Langmuir constant, *C*_s_ is the molar concentration of the standard reference solution (1 mol L^–1^), and γ_e_ is the activity coefficient calculated using the Davies equation at an ionic strength of 0.025 M.^63^

### Aqueous V^V^ Speciation

Visual MINTEQ version 3.1 was used to calculate the V^V^ speciation for each equilibrium V^V^_aq_ concentration obtained in the Langmuir isotherm experiments. Parameters for the calculation included 25 mM NaCl of background electrolyte, and the pH was fixed at 7 to account for the 10 mM of PIPES buffer. Results are presented in Section III of the Supporting Information (SI).

### X-ray Absorption Spectroscopy

X-ray absorption spectra were collected on all samples with initial V^V^ concentrations of 1.5 mM, 100 μM, and 50 μM. These concentrations were selected for EXAFS measurement on the basis of detector limitations, reports of comparable concentrations at contaminated sites,^25^ and to test for the presence of adsorbed polyvanadate species. The XAS measurements were conducted at the Stanford Synchrotron Radiation Lightsource. All samples were sealed in 13 μm thick Kapton tape. Room temperature vanadium K-edge EXAFS were collected at beamline 4–3 with a He purge box to reduce oxygen infiltration (O_2_ < 0.15%). Spectra were collected from 5235 to 6300 eV in fluorescence mode using a seven-channel Si drift detector (Canberra) and energy selection provided by a Si(111) crystal set oriented to φ = 90°. After each scan, the samples were moved vertically by 1 mm to avoid beam-induced photoreduction. An in-line V^0^ foil was used for energy calibration by setting the peak of the first derivative to 5465 eV. Background subtraction and normalization was performed using Athena software (Windows v9.26).^64^

Nonlinear least-squares shell-by-shell fitting was performed using Artemis as an interface to Feff6 and IFEFFIT.^64^*E*_0_ was set at the value of the absorption edge inflection point (∼5482 eV) for each spectrum. The k^3^-weighted χ(k) data were Fourier transformed using a sine windowing function to acquire the pseudoradial structure function. Backscattering paths were then fit to the transformed data using multiple *k*-weighting to derive relevant interatomic distances and coordination numbers. The distribution of expected aqueous V^V^ species was calculated using Visual MINTEQ 3.1 (SI Section III) and were used to inform the shell-by-shell modeling of EXAFS spectra. Additionally, prior studies using EXAFS to characterize vanadate adsorption by ferrihydrite and goethite provided a baseline for comparison to the data in this study.^34,39,40^

Mn K-edge XAS spectra were also collected to assess any transformation to birnessite by the PIPES buffer. This data is presented in SI Section V.

## III Results

### Isotherm Modeling

**Figure 1:**
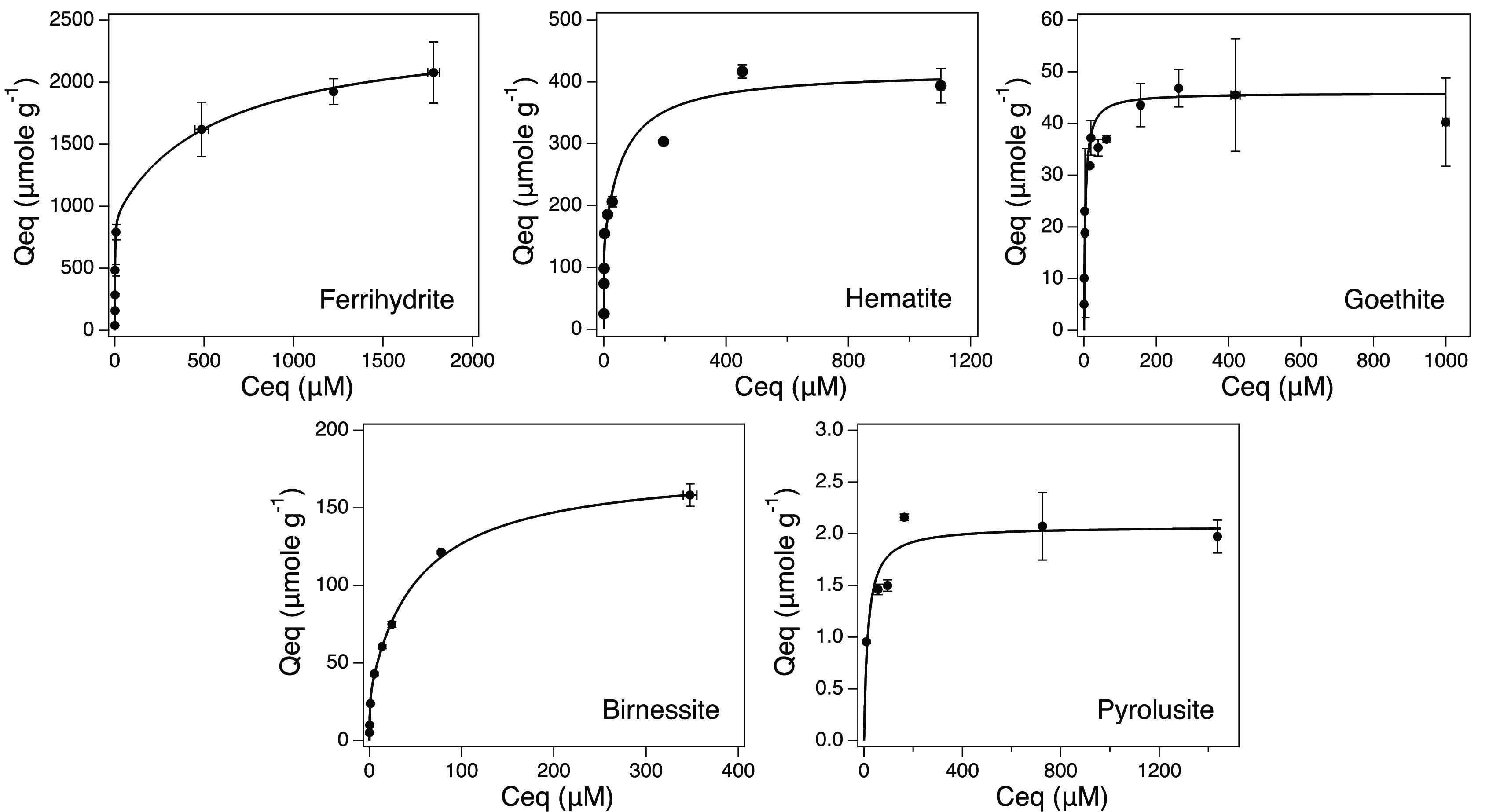
Plots of the aqueous equilibrium V^V^ concentration (μM) vs the adsorbed V^V^ (μmole g^–1^). Note that the scale of *x* and *y* axes varies from plot to plot.

Across all sorbents, the observed *C*_eq_ values ranged in value by 6 orders of magnitude. The isotherm results are presented in Figure 1, and each isotherm is characterized by a steep initial slope and a plateau characteristic of H-type isotherms.^55^

The suitability of the Langmuir model to the data is confirmed by a linear relationship between *C*_eq_ and  (Figure S3). Deviations from this linearity at low concentrations indicate the saturation of a small proportion of high-affinity sites, which requires the application of the 2L model to accurately obtain model parameters *q*_max_ and *K*_L_. The resulting values of *q*_max_ and *K*_L_ obtained from the model are reported in Table 1.

**Table 1:** Fit Parameters Obtained *via* NLLS Regression of the *C*_eq_ vs *q* Data Using a Two-Site Langmuir Model^a^

		site 1	site 2	site 1	site 2	site 1	site 2	
	surface area (m^2^ g^–1^)	*q*_max_ (mol g^–1^)		ln(*K*_L_) (L mol^–1^)		Δ*G*°_ads_ (kJ mol^–1^)		ratio of high to low Δ*G*°_ad_
ferrihydrite	176.90	9.18 × 10^–04^	1.52 × 10^–03^	13.70	7.46	–33.96	–18.67	1.82
hematite	61.29	2.85 × 10^–04^	1.34 × 10^–04^	9.71	15.99	–24.18	–39.56	1.64
goethite	28.87	2.94 × 10^–05^	1.65 × 10^–05^	12.09	13.15	–30.01	–32.60	1.09
birnessite	37.80	3.07 × 10^–05^	1.47 × 10^–04^	14.78	9.85	–36.61	–24.52	1.49
pyrolusite	1.16	2.07 × 10^–06^		11.05		–27.47		

	site 1	site 2	site 1	site 2	goodness of fit	
	half-saturation concentration (mole L^–1^)		(sites nm^2^)		RMSE	*R*^2^
ferrihydrite	1.12 × 10^–06^	5.74 × 10^–04^	3.12	5.19	181.97	0.972
hematite	6.06 × 10^–05^	1.14 × 10^–07^	2.80	1.31	43.98	0.937
goethite	5.62 × 10^–06^	1.95 × 10^–06^	0.61	0.34	5.17	0.899
birnessite	3.81 × 10^–07^	5.26 × 10^–05^	0.49	2.34	1.70	0.999
pyrolusite	1.58 × 10^–05^		1.07		0.22	0.810

a *Q*_max_ is the maximum adsorption capacity for a given site; *K*_L_ is the Langmuir constant; *K* is the dimensionless equilibrium coefficient; Δ*G*°_ads_ is the free energy of adsorption; and the RMSE and *R*^2^ are goodness of fit parameters

As expected, V^V^ showed greater retention on the oxide phases with lowest crystallinity (Birn and Fhy). When normalized for surface area, Fhy and Birn were found to contain more sites per nm^2^ than their more crystalline counterparts, with overall site density following the order of Fhy > Hm > Birn > Gt > Pyr. Hematite and Fhy were found to have >1 site nm^–1^ for both high- and low-affinity sites, while among the Mn oxides, only birnessite’s low-affinity site had a density of >1 nm^–1^.

In a side-by-side comparison of the linearized Langmuir isotherms, the steeper slope of the Pyr isotherm is indicative of a low-affinity interaction across the entire range of V^V^ loading (Figure S3). Unlike the other oxides, a one-site Langmuir model was sufficient to model the data, with the addition of a second site consistently resulting in a *q*_max_ of 0 when the 2L model was applied. The application of a one-site model revealed that Pyr had an abundance of 1.07 sites nm^2^ that reached half-saturation at 15.8 μM *C*_eq_, corresponding to a surface loading of 1.04 μmole g^–1^, whereas Birn exhibits a steep slope at low *C*_eq_, which inflects to a shallower slope after 13 μM *C*_eq_. This suggests that Birn contains a relatively small number of high-affinity sites, which become saturated below this concentration threshold. Applying the half-saturation formalism described by Sugihara et al.,^65^ the *C*_eq_ corresponding to half-saturation for the high-affinity sites on Birn is actually found to be much lower (∼0.4 μM; Table 1), while 13 μM *C*_eq_ corresponds to approximately 12% of the total low-affinity site coverage.

The Gt Scatchard transformation shows that two distinct site types are present (Figure S2). Though the linearization of the Langmuir function results in a good fit (*R*^2^ = 0.997), the Scatchard transformation shows a steep initial descending slope suggestive of high-affinity sites, which are saturated when *q*_eq_ = 32 μmole g^–1^ (corresponding to a *C*_eq_ of 16 μM). The half-saturation concentration of Gt’s high-affinity site (1.9 μM) corresponds to the rising edge of the isotherm with the half-saturation of the low-affinity site occurring when *C*_eq_ = 5.6 μM.

Hematite, which is highly crystalline (Figure S1), retained more V per gram of oxide than Gt (Table 2), likely due to the smaller particle size and higher surface area. The linearized Langmuir plot (Figure S3) for V sorption on Hm displays an inflection point similar to the results that observed low V^V^ concentrations on Birn. This, along with the Scatchard transformation, affirms the need for a two-site model. The high-affinity site on Hm has the highest affinity for V^V^ at low concentrations of all oxides examined, reaching half-saturation when *C*_eq_ is only 100 nM. However, the low-affinity sites of Hm have much worse affinity than the low-affinity sites of Gt (Table 1); this indicates that the higher *q*_max_ of Hm is due to its larger surface area compared to Gt.

**Table 2:** Results from Nonlinear Least Squares Shell-by-Shell Fitting of the V K-edge EXAFS^a^

sample	CN	*R* (Å)	σ^2^ (Å^2^) x10^–3^	*S*_0_^2^	Δ*E*	K range	R-factor	**χ**^2^_red_
ferrihydrite								
1.5 mM V(V)								
V–O	2.2 (2)	1.66 (2)	0.9 (5)					
V–O	1.9 (2)	1.80 (3)	0.9 (5)	0.9 (1)	–4 (5)	3–12.5	0.008	30.6
V–O–O	12	3.174 (5)	1.7 (9)					
V–Fe	1	2.78 (5)	14.5 (5)					
V–Fe	2	3.33 (8)	22 (9)					
100 μM V(V)								
V–O	2.1 (3)	1.66 (2)	1.0 (4)					
V–O	1.9 (3)	1.79 (2)	1.0 (4)	0.72 (6)	–3 (1)	3–11	0.006	36.4
V–O–O	12	3.124 (4)	1.8 (8)					
V–Fe	1	2.78 (8)	20 (10)					
50 μM V(V)								
V–O	2.1 (3)	1.66 (2)	2 (2)					
V–O	1.9 (3)	1.78 (2)	2 (2)	0.81 (7)	–3 (2)	3–12.5	0.001	5.2
V–O–O	12	3.16 (2)	3 (3)					
V–Fe	1	2.78 (2)	18 (4)					
V–Fe	2	3.35 (2)	21 (4)					
goethite								
1.5 mM V(V)								
V–O	2.0 (8)	1.65 (6)	0.9 (6)					
V–O	2.0 (8)	1.78 (7)	0.9 (6)	0.8 (1)	–4 (8)	3–11.5	0.007	41
V–O–O	12	3.174 (6)	2 (1)					
V–Fe	1	2.8 (1)	20 (10)					
V–Fe	2	3.29 (7)	16 (8)					
100 μM V(V)								
V–O	2.0 (2)	1.67 (2)	1.0 (3)					
V–O	2.0 (2)	1.79 (2)	1.0 (3)	0.83 (5)	–1 (2)	3–12.5	0.005	7.8
V–O–O	12	3.174 (3)	1.9 (5)					
V–Fe	1	2.77 (3)	16 (4)					
V–Fe	2	3.37 (2)	13 (2)					
50 μM V(V)								
V–O	1.7 (3)	1.65 (2)	1.0 (2)					
V–O	2.3 (3)	1.77 (1)	1.0 (2)	0.90 (5)	–3 (2)	3–12.5	0.001	13.3
V–O–O	12	3.16 (3)	1.8 (5)					
V–Fe	1	2.79 (3)	18 (5)					
V–Fe	2	3.37 (3)	17 (3)					
hematite								
1.5 mM V(V)								
V–O	2.0 (5)	1.62 (5)	1.0 (5)					
V–O	2.0 (5)	1.76 (6)	1.0 (5)	0.7 (2)	–9 (8)	3–12	0.011	51.5
V–O–O	12	3.174 (5)	1.8 (9)					
V–Fe	1	2.66 (4)	14 (6)					
V–Fe	2	3.38 (9)	14 (6)					
100 μM V(V)								
V–O	2.1 (5)	1.67 (3)	1.0 (4)					
V–O	1.9 (5)	1.79 (3)	1.0 (4)	0.88 (5)	–2 (2)	3–11	0.002	25.1
V–O–O	12	3.17 (3)	1.8 (7)					
V–Fe	1	2.79 (3)	17 (5)					
V–Fe	2	3.40 (3)	19 (4)					
50 μM V(V)								
V–O	1.7 (3)	1.65 (2)	0.9 (4)					
V–O	2.3 (3)	1.77 (2)	0.9 (4)	0.93 (6)	–3 (2)	3–12.5	0.002	32.4
V–O–O	12	3.17 (3)	1.7 (7)					
V–Fe	1	2.77 (3)	15 (3)					
V–Fe	2	3.37 (3)	17 (3)					
birnessite								
1.5 mM V(V)								
V–O	2.6 (1)	1.63 (2)	5.0 (2)	0.71(1)	–9(1)	3.5–11	0.008	20
V–O	1.4 (1)	1.79 (4)	5.0 (2)					
V–O–O	12	3.174 (5)	9.3 (5)					
100 μM V(V)								
V–O	2.3 (1)	1.63 (2)	1.1 (2)					
V–O	1.7 (1)	1.80 (3)	1.1 (2)	0.72 (8)	–7 (6)	3.5–12.5	0.014	4.2
V–O–O	12	3.16 (5)	2.0 (4)					
V–Mn	1	2.65 (4)	14 (4)					
50 μM V(V)								
V–O	2.4 (1)	1.63 (2)	3.1 (4)	0.71 (2)	–8 (1)	3.5–12	0.011	9.2
V–O	1.6 (1)	1.77 (3)	3.1 (4)					
V–O–O	12	3.13 (4)	5.7 (8)					
pyrolusite								
100 μM V(V)								
V–O	1.5 (2)	1.61 (2)	1.0 (2)					
V–O	2.5 (2)	1.74 (3)	1.0 (2)	0.70 (7)	–6 (3)	3–11.5	0.004	3
V–O–O	12	3.124 (2)	1.8(3)					
V–Mn	1	2.76 (4)	18 (2)					
50 μM V(V)								
V–O	1.9 (9)	1.65 (5)	1.0 (3)					
V–O	2.1 (9)	1.77 (5)	1.0 (3)	0.72 (9)	–3 (4)	3–11.5	0.003	28.7
V–O–O	12	3.15 (5)	1.8 (6)					
V–Mn	1	2.79 (7)	18 (3)					
V–Mn	2	3.35 (7)	20 (10)					

a CN is the coordination number, *R* is the interatomic distance in Å, σ^2^ is a measure of the static and thermal disorder for each coordinating interatomic path, Δ*E* is a shift parameter to align the EXAFS theory with the data, and *S*_0_^2^ is the amplitude reduction term.

Two-line Fhy exhibited the highest *q*_max_ of any oxide, as well as having the largest surface area (Table 1). Like for Gt, Langmuir linearization resulted in a good fit (*R*^2^ = 0.995), though the Scatchard plot again suggests that multiple sites are required to model the Fhy-V^V^ isotherm. The inflection between the rising edge and asymptotic portions of the isotherm occur between *C*_eq_ of 6.5 and 486 μM. The Fhy high-affinity sites reach half-saturation at *C*_eq_ of 1.1 μM, corresponding to a surface coverage of ∼480 μmole g^–1^, while Fhy low-affinity sites reach half-saturation at *C*_eq_ of 573 μM, corresponding to *Q*_eq_ of ∼1700 μmole g^–1^. Thus, at C_eq_ of 6.5 μM, the high-affinity sites are expected to be fully saturated.

### Thermodynamic Calculations

Thermodynamic parameters calculated for each V^V^–sorbent interaction are provided in Table 1. When site affinity is calculated as a function of Δ*G*°_ads_, the ratio of high-affinity to low-affinity sites across oxides follow the order observed for site density. Notably, Hm, Fhy, and Birn have affinity ratios of ∼1.5 or greater (Table 1), while Gt is approximately isoenergetic between the two sites (∼1.09 kJ mol^–1^). This suggests that the surface site-type distribution of V^V^ on Gt is relatively homogeneous compared to Birn, Fhy, and Hm. This is likely a result of greater surface heterogeneity on Birn, Fhy, and Hm arising from a larger range of truncating hkl surfaces, particularly for Hm^66^ relative to the predominance of the (110) plane at the Gt surface.^67,68^ While a site affinity ratio could not be calculated for pyrolusite, it is likely comparable to Hm given the variety of hkl planes present at the oxide surface.^69^

### Results from EXAFS

**Figure 2:**
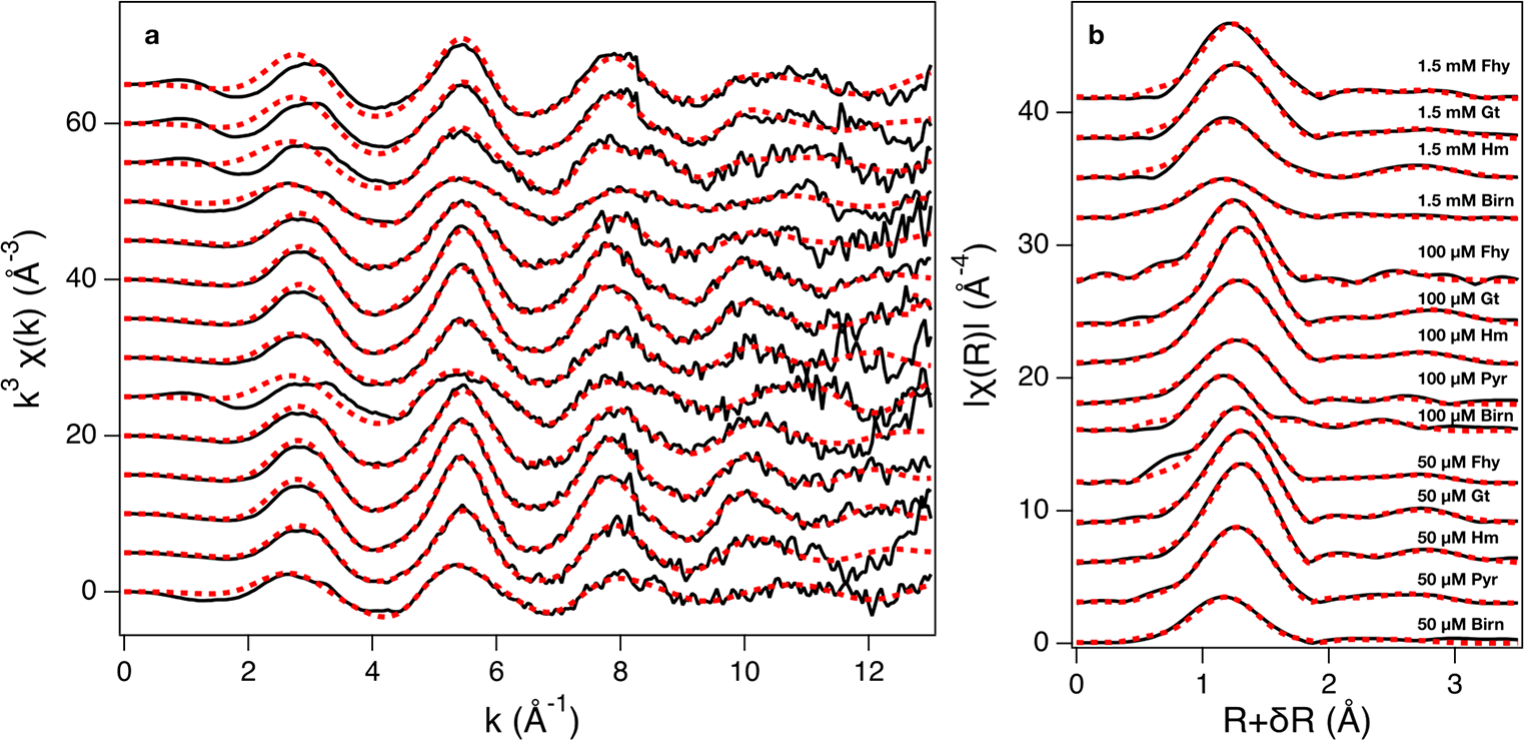
k^3^-weighted V K-edge EXAFS of V^V^ adsorbed on ferrihydrite (Fhy), hematite (Hm), goethite (Gt), birnessite (Birn), and pyrolusite (Pyr) at 50 μM, 100 μM, and 1.5 mM initial V^V^ concentrations. (b) Pseudoradial structure function of the EXAFS. For consistency, samples are arranged identically in each panel.

The results of the fitting are presented in Table 2 and Figure 2. Coordination numbers were constrained to the crystallographic values of vanadate for the V–O single scattering and intratetrahedral multiple scattering paths and to the expected V-Me values for a given type of surface complex. Vanadium EXAFS spectra of the pyrolusite incubated with 1.5 mM V^V^ could not be collected due to the high crystallinity and large particle size of pyrolusite, which caused excessive elastic scattering of the incident X-rays that saturated the detector even after additional pulverization. While both bidentate-binuclear corner sharing complexes (^2^C) and bidentate mononuclear edge sharing complexes (^2^E) were observed between vanadate and the Fe oxides, vanadate primarily forms ^2^E complexes on Mn oxides.

## IV Discussion

### Iron Oxides

The free energy of adsorption, Δ*G*°_ads_ was negative for all observed interactions, indicative of thermodynamically spontaneous processes. Hematite and Gt exhibited similar Δ*G*°_ads_ for V^V^, while the Δ*G*°_ads_ of Fhy was ∼20% lower, which demonstrates that affinity increases with crystallinity, as has been observed in other adsorption systems.^31^ A similar relationship has been observed for the adsorption of As^III^ on these minerals despite the typically anhydrous nature of Hm, which is likely attributable to their ability to accommodate similar modes of adsorption.^67^ For the Hm used in this study, the ratio of the intensities of the (104) and (113) peaks was ∼3.1, where a ratio of ∼4 would be indicative of pure anhydrous α-Fe_2_O_3_.^50^ This indicates that our Hm is partially hydrated, with Fe vacancies to balance the presence of H^+^ and a partially hydroxylated surface that can better accommodate the adsorption of the V^V^ oxyanion along the (001) and (110) faces.^67,70−72^ In contrast, the (110) face of Gt is both the most abundant face and is also hydroxylated, which supports oxyanion adsorption with minor contributions from the (101) face.^67,68,73,74^

The difference in site affinity between the Gt and Hm can be described by differences in their points of zero charge (pzc). The pzc for Gt typically ranges between 7.5 and 9.5; a pzc of 8.5 was used in a past study modeling the adsorption of vanadate by Gt.^34,72,75^ The pzc of Hm is generally slightly higher than that of Gt ranging from 8.4 to 9.4.^72,75^ The higher pzc of Hm results in increased attraction for the H_2_VO_4_^–^ anion (p*K*_a1_ = 7.91,^76^ p*K*_a2_ = 8.06,^12^ p*K*_a3_ = 8.8^7^), which is the predominant V^V^_aq_ species when V concentrations are <1.5 mM at pH 7 (SI Section III). Additionally, imperfections on the (001) and (110) faces of Hm because of Fe vacancies and hydration increase the pzc. This leads to long singly coordinated Fe–O bonds at the Hm surface, resulting in pzc values of up to 11 for select faces.^72^

Due to the predicted presence of polyvanadate species at all but the lowest concentrations tested, it remains a possibility that Hm can retain highly charged polymeric vanadate species more efficiently than Gt. For example, Peacock and Sherman^34^ noted a decreased retention of V by Gt at pH 7 at concentrations high enough for polymeric V formation when compared to systems with lower V concentrations. A combination of *ab initio* modeling and EXAFS measurements were used to investigate the types of surface complexation present at V concentrations of 50 and 500 μM, with exclusively ^2^C complexes reported over a pH ranged of 2.85 to 8.9.^34^ The authors argued that the formation of ^2^E complex should not be considered on the basis of modeled energetic favorability and the *a priori* requirement that multiple scattering paths should be included in FEFF calculations, where a failure to do so would result in spurious detection of the ^2^E complex. However, another study of vanadate adsorption on ferrihydrite utilized multiple scattering and wavelet transform (WT) analysis,^39^ concluding that a ^2^E Fe^III^(O,OH)_6_-vanadate complex exists for ferrihydrite, with the vanadate tetrahedron distorted into an approximately square-planar geometry. The results supported the conclusion that multiple scattering within the vanadate tetrahedron hides the EXAFS contribution from the Fe in the second shell of the EXAFS pseudo-RSF plot, and hence, no ^2^C complex was reported. Later work by Vessey and Lindsay^40^ corroborated these results for ferrihydrite. However, whereas Larsson et al. had included several multiple scattering paths to improve the fit that went beyond the intratetrahedral V–O–O path at ∼3.15 Å (including V–O–Fe and V–O–O hinge/rattle paths between 3 and 4 Å), Vessey and Lindsay were able to fit a ^2^C V–Fe complex and reported both ^2^E and ^2^C V–Fe distances that were comparable to the *ab initio* calculated V–Fe distances of Peacock and Sherman.^34,39,40^

In our analysis, both ^2^E and ^2^C complexes were observed for Fhy, Gt, and Hm. Twelve intratetrahedra V–O–O MS paths were included in all fits and did not interfere with the detection of the ^2^E complex, as demonstrated in Larsson et al.^39^ Further, our observed V–Fe distance for the ^2^E complex on Fhy was similar to that reported by Larsson et al.^39^ at 2.78 Å. This value is longer than that reported by Vessey and Lindsay, which may be due to the lower ionic strength employed by our study (25 mM) and by Larsson et al. (10 mM)^39^ as compared to Vessey and Lindsay^40^ (50 mM). A lower IS leads to an increased thickness of the Stern layer at the mineral surface, which can affect vanadate adsorption modes.^77^

Our study agreed with the conclusion of Peacock and Sherman^34^ and Larsson et al.^39^ regarding the importance of the MS paths to the quality of the fit. Specifically, the 12 intratetrahedral V–O–O paths at 3.12–3.17 Å were found to be most important. While Peacock and Sherman described the importance of the various MS paths, they did not report the fitted half-path lengths nor the σ^2^ values for these paths, which made comparisons difficult. Given the distance resolution of EXAFS allowable by eq 4,
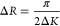
4the intratetrahedral MS paths ranging can be difficult to distinguish from the ^2^C V–Fe distance of ∼3.30 Å unless a Δ*k* > 10 is used, which is difficult to achieve at low surface loadings. Similarly, the V–O–Fe path for the ^2^E complex, while a weak contributor to the overall EXAFS signal, can occur from 3.19 to 3.25 Å, further interfering with the detection of the ^2^C complex unless conditions are ideal. One method that has been used to resolve such interferences is by fitting the EXAFS spectra with multiple k-weights simultaneously, which will amplify specific segments of the spectrum. For example, MS paths are typically strongest at low values of *k* and thus are most emphasized by low *k*-weighting, while single-scattering paths are present throughout the spectrum and can be amplified with higher k-weights. When this approach was applied in this study, the results for V adsorption on Fhy are comparable to that presented by Larsson et al.^39^ However, we did not observe an improvement to the fit with the inclusion of further MS paths. Iterations of our fits included all combinations of ^2^E, ^2^C, and ^2^E V–O–Fe at 3.25 Å, ^2^C V–O–Fe at 3.50 Å, and intratetrahedral hinge and rattle V–O–O contributions at 3.45 Å. Further, we found that the 4 + 12 V–O–O rattle and hinge paths at 3.45 Å described by Larsson et al.^39^ occur predominately in the Na_3_VO_4_ crystal structure often used as a source of V–O and V–O–O paths for V^V^ EXAFS. As such, we do not expect them to occur in a surface complex with any of the minerals tested.

Like Larsson et al.^39^ and Vessey and Lindsay,^40^ we observed relatively large Debye–Waller factors for the ^2^E V^V^–Fe complex, which may be a result of heterogeneity in the V binding environment. Although we also observed large Debye–Waller factors for the V^V 2^C complexes as well, we attribute this to data having been collected at room temperature and a relatively short Δ*K* range refined in the fitting due to degradation of the data quality at values of *k* > 11.5 Å ^–1^ in many cases.

Inner-sphere adsorption of polymeric oxometallates has been observed at the surface of Hm for polyvanadate and polytungstate.^31,78^ Specifically, Hm appears to effectively retain H_2_V_2_O_7_^2–^ and V_4_O_12_^4–^ at its surface.^31^ These species constitute ∼5% of total V^V^_aq_ below 100 μM and ∼30% below 1000 μM (SI Section III). Hematite’s affinity for these polyvanadate species has been shown to be greater than that of Fhy, due to favorable interactions at the (001) surface.^31^ This serves to explain why Fhy bore a higher affinity for V in our study, as the Hm used here exhibited minimal basal (001) character despite a low amount of platy morphology implied by the presence of a small (006) peak in the powder XRD pattern; instead, surfaces appear to be dominated by the (024), (104), (110), and possibly (014) faces (Figure S1).^66,79^ As noted by Venema et al.^72^ and Ona-Nguema et al.,^67^ singly coordinated reactive oxygens at the (110) surface are ideal for supporting ^2^C surface complexes and are the most likely candidate for hosting adsorbed monomeric V in the ^2^C configuration. The ratio of singly coordinated O to doubly coordinated O of the Fe face-sharing octahedra is 2:1 per unit cell. While ^2^E complexes may form at this face and the (001) face, the (001) face is predicted to have three singly coordinated O atoms per unit cell forming the face of a single Fe octahedron, making adsorption more favorable than at the (110) face due to the lower reactivity of the doubly coordinated O atoms at that face.

Ferrihydrite displayed the greatest adsorption capacity for V^V^. This is a function of its high surface area, site density, Fe vacancies, and abundant singly- and doubly coordinated surface −OH groups.^39,67,80^ The pH_pzc_ for two-line Fhy ranges from 7 to 8;^81^ therefore, the Fhy surface holds a slight positive charge at pH 7 and is expected to electrostatically attract aqueous V^V^ anions. Given the high degree of disorder in the stacking of Fhy lattice planes,^82^ the formation of the ^2^E complex with vanadate reported previously^39^ and in this work likely occurs at the (100) surface.^67^ Several structural motifs have been reported for two-line Fhy including a maghemite-like structure, a hexagonally stacked double-chain like structure, a Baker-Figgis δ-Keggin-like cluster, and closely packed anionic sheets with high amounts of stacking disorder and interlayer Fe.^80,82,83^ Thus, it is difficult to determine which hkl surface is most favorable for V^V^ adsorption; however, it is likely that the presence of Fe vacancies and relative higher abundance of singly coordinated O atoms make the (100) and (010) faces more likely to adsorbed V^V^ than the (001) face.^80,83^ In the model proposed by Michel et al.^83^ the (100) and (010) faces host doubly- and singly coordinated O in a 7:1 ratio with equivalent positions for ^2^E or ^2^C adsorption. However, the relative abundance of (100) to (010) faces is difficult to determine without further study, and our bulk XRD measurements only confirm the abundance of (100) planes throughout the structure of our Fhy.

It is possible that the high capacity of Fhy for V^V^ is due to polymeric V complexation. While observed for other polymerizing d-block elements (Mo, W) on Hm,^78,84^ the paucity of comparable observations for polymeric V on Fhy is likely due to the low concentrations typically examined (≤100 μM).^31,39,77^ However, recent evidence supports the retention of tetrahedrally coordinated polyvanadate species such as pyrovanadate (V_2_) but not octahedrally coordinated V^V^ species such as decavanadate (V_10_) on Fhy.^31^ Unlike Mo,^84^ epitaxial and surface-catalyzed growth of the polyvanadate species is not expected under the conditions examined in the present study due to the rapid kinetics of polymeric V^V^ formation,^85^ and steric hindrance due to the large size of decavanadate.^31^ Thus, it is likely that the process of V_2_ complexation is an adsorption process as opposed to surface-catalyzed polymerization.

The limitations of EXAFS also complicate the resolution of polyvanadate surface species that can be resolved. It is difficult to distinguish between atoms of similar atomic number using EXAFS, and thus it can be challenging to discern between V and Fe at a similar half-path length.^86^ However, the bonding of V_2_ as pyrovanadate to an octahedrally coordinated manganese has been detected with EXAFS in the context of structural biology.^87^ Finally, an examination of the ^2^C distances obtained for the 1.5 mM V^V^ treatments of Fhy and Gt reveal V–Fe distances of 3.29 to 3.33, which are comparable to previous studies;^34,40^ however, the ^2^C distance for V^V^ on Hm was 3.38 Å. While it is possible that the V–Fe distance is greater with Hm than Fhy or Gt, 3.38 Å is also the average between a V–Fe ^2^C distance at 3.33 Å and the crystallographic V–V distance of pyrovanadate at 3.42 Å. Given the difficulty in distinguishing between V and Fe by EXAFS, it is likely the resultant distance is contributed by both backscattering atomic pairs, which provides evidence for polyvanadate retention by Hm.

### Manganese Oxides

Vanadium (V) retention on Mn oxides increased as a function of decreasing crystallinity similar to adsorption of V^V^ on Fe (oxyhydr)oxides, with more exergonic V^V^ adsorption on Birn than on Pyr. Although methods used to calculate estimates of the dimensionless equilibrium coefficient (*K*) and Δ*G*°_ads_ from the Langmuir constant have been rigorously established,^58,63,88,89^ some variability between methods exists (primarily in the derivation of *K*_L_ from a linearized Langmuir equation).^88^

The Δ*G*°_ads_ for the low-energy site of Birn was approximately equal to that of the high-energy site on Pyr. The lower affinity of Pyr for V^V^ adsorption is likely due to the anhydrous nature of the bulk mineral. While the formation of an amorphous, hydrous layer has been reported in nanophase Pyr when solvated by water, no such amorphous MnOOH formation has been reported for the surface of bulk-phase Pyr.^90^ Therefore, the number of singly coordinated O and reactive −OH groups available on Pyr is expected to be low, which likely explains the low amount of V^V^ retained. This hypothesis is further supported by bond valence calculations yielding high degrees of terminal O bond saturation in Pyr relative to two-line Fhy and Birn.^81^ This leads to lower likelihood that terminal O at the Pyr surface will be sites for inner-sphere V adsorption. Furthermore, Pyr has been reported as having a higher surface energy than Birn,^90,91^ which leads to a stronger retention of water in the hydrating layer. This results in a greater energy barrier for the displacement of water in this layer by V^V^ in the formation of an inner-sphere complex. In the 100 μM V^V^–Birn incubation, the ^2^E V–Mn distance was 2.65 Å, compared to 2.76 Å with Pyr. However, the V EXAFS of solids from 50 μM and 1.5 mM V^V^–Birn incubations did not reveal a distinct V–Mn peak. This may indicate the formation of outer-sphere complexation at the birnessite surface, given that only contributions from the coordinating oxygen and V–O–O MS paths were observed. The χ(k) data for these samples resembles EXAFS analysis of selenate sorption on goethite^92^ which were similarly attributed to outer sphere complexation due to a lack of Se–Fe backscattering contributions. Only one prior study has looked at vanadate associated with birnessite using V EXAFS,^93^ which examined how birnessite synthesized with various degrees of V^V^ doping would affect the oxide’s ability to scavenge metal cation contaminants. They described a surface coating of V_6_O_16_^2–^ hexameric vanadate polymers, yielding possible V–Mn distances of 2.97–3.06 and 3.43–3.5 Å, much longer than what was observed in this study.

Synthetic Pyr generally has a higher pH_pzc_ (5.98 to 4.3) than Birn (∼2–4),^94−97^ and a much higher pH_pzc_ for Birn edge sites has been proposed (6–7).^81,95,98^ At pH 7, the surface of Pyr is thus expected to be dominated by negatively charged, saturated oxygens, which can electrostatically repel H_2_VO_4_^–^. As a result, we attribute the formation of ^2^C and ^2^E complexes on Pyr to the (110) and (100) faces. ^2^C complexes should form more favorably due to the solvent-facing orientation of the singly coordinated O at these faces. In contrast, reactive terminal hydroxyl groups on Birn are available for inner-sphere adsorption and ligand exchange. This also explains why no V^V^ adsorption moieties were observed at the Birn layer vacancies within the *ab* plane, as these are a source of negative layer charge that contributes heavily to the low pH_pzc_ of the bulk Birn, repelling anionic V^V^.^95,99^

The results from the EXAFS corroborate the expected sorption affinities predicted by a comparison of the mineral pzc values. The peak corresponding to the nearest Mn neighbor in the pseudo-RSF plot is markedly lower in amplitude for the Pyr samples than it is for results from 100 μM V^V^–Birn incubation, reflecting a lower surface loading (Figure 2). However, σ^2^ values for the V–Mn paths are greater for Pyr than for Birn (Table 2). This could be due to measurements being conducted at room temperature as well as the perturbation of the Pyr surface by hydration leading to heterogeneous bonding environments. Hydration-induced perturbation of the surface of nano-Pyr has been reported previously using XRD as the primary method of detection.^90^ However, the low surface area of bulk-phase Pyr made any such perturbations below the detection limit in our study. A hydrated amorphous phase developing at the Pyr surface would be situated ideally to interact with adsorbents such as the V^V^ examined here, which is theoretically measurable with EXAFS. Thus, competition for aqueous vanadate between a discontinuous, amorphous surface Mn phase and exposed faces of unreacted Pyr, taken in conjunction with the room temperature environment for the EXAFS measurements may explain the relatively large σ^2^ and interatomic distance error. Future studies probing the alteration of the surface of macro-crystalline Pyr by hydration and its resulting effects on adsorption are needed to verify this hypothesis. Finally, we were unable to account for the extent of outer sphere complexation in the retention of V by Pyr due to the limited resolution of the V EXAFS at the concentrations examined.

## V Conclusions

Previous studies examining the interactions of aqueous V^V^ at the water–solid interface have focused primarily on Gt and Fhy^34,39^ and rarely discuss the role of polynuclear V^V^ species in adsorption.^31^ The present study examined the adsorption of V^V^ on several common Fe (oxyhydr)oxides and Mn (hydr)oxides using an isotherm approach paired with EXAFS to determine uptake affinities and coordination geometries. While mononuclear V^V^ was the only species detected *via* EXAFS at and below 100 μM V^V^, evidence for polyvanadate adsorption could be detected with 1.5 mM V^V^. The ability of V^V^ to adsorb onto Fe (oxyhydr)oxide and Mn (hydr)oxide octahedra implies that competitive adsorption–desorption interactions will occur in the presence of common oxyanions such as phosphate, as well as coexisting contaminants such as arsenate. On the basis of our findings and the relative of abundance of Fe oxides relative to Mn oxides, we expect Fe oxides to be the dominant sorbent phase for vanadate in oxic terrestrial systems with available surface area being a key factor in vanadate retention. In conclusion, the exergonic adsorption of V^V^ onto both the Fe and Mn (hydr)oxides examined suggests that V adsorption is thermodynamically favorable for a range of surfaces that display differing levels of hydration and Fe/Mn–O bond saturations. The description of these reactions using Langmuir adsorption parameters situates these results for use in distribution and transport modeling for more accurate predictions of V partitioning at the solid–solution interface.

## Supporting Information Available

Discussions of mineral characterization, aqueous V(V) speciation, method for nonlinear least squares fitting, method for birnessite characterization, and mineral synthesis, figures of results of BET surface area analysis, results of BJH porosity analysis, XRD reflection, scatchard plots, linearized langmuir plots, MINTEQ speciation results, and Mn K-edge XAS, and table of MINTEQ model results (PDF)
