# Supplementary material for: Vanadate Retention by Iron and Manganese Oxides
Source: ACS Earth Space Chem. 2022 Aug 5;6(8):2041–52. doi: 10.1021/acsearthspacechem.2c00116 (PMC9393891; doi:10.1021/acsearthspacechem.2c00116)
Supplement: Supplementary file 1 — sp2c00116_si_001.pdf [file sp2c00116_si_001.pdf]

Supporting Information

Vanadate retention by Iron and Manganese Oxides

Macon J. Abernathy[1], Michael V. Schaefer[2], Roxana Ramirez[3], Abdi Garniwan[3], Ilkeun Lee[4], Francisco Zaera[4], Matthew Polizzotto[5] and Samantha C. Ying[1,3*]

[1]Stanford Synchrotron Radiation Lightsource, SLAC National Accelerator Laboratory, Menlo Park, CA 94025, USA

[2]Department of Earth and Environmental Science, New Mexico Institute of Mining and Technology, Socorro, NM 87801, USA

[3]Environmental Sciences Department, University of California-Riverside, Riverside, CA 92521, USA

[4]Department of Chemistry, University of California-Riverside, Riverside, CA 92521, USA

[5]Department of Earth Sciences, University of Oregon, Eugene, OR 97403, USA

*Corresponding Author: *E-mail address*: samying@ucr.edu

Table of Contents

Supporting Information ***1***

Section I. Mineral Characterization **3**

Section II. Transformed Isotherms **11**

Section III. Aqueous V^V Speciation **13**

Section IV. Method for Nonlinear Least Squares Fitting **16**

Section V. Method for Birnessite Characterization **18**

Section VII. Mineral Synthesis **20**

Birnessite 20

Hematite 20

2-line Ferrihydrite 20

Section VIII. References **21**

Section I. Mineral Characterization

The properties of the Fe and Mn oxides used in sorption experiments are presented in Table 2.1. XRD analysis confirmed the identity of each mineral phase (Figure S1c-g). Birn was synthesized with potassium in the interlayer space, matching previously reported hkl reflections [1]. The hexagonal symmetry of the Birn was verified by the ratio of d-spacings for the (11,20) and (31,02) reflections approximating $\sqrt{3}$ with a value of 1.69 [2–4]. Further, the (31,02) reflection is symmetric and lacks the splitting indicative of orthogonal symmetry [2]. The (001) reflection was used to calculate the d_{001} and the coherent scattering domain (CSD) along the c* axis, which were 7.2 Å and 6.4 nm respectively and are consistent with previously reported d_{100} values [3] and 8.8 coherently stacked layers along the c* axis [2,3,5,6]. The CSD of the ab plane was calculated to be 6.1 nm from the (11,20) peak, in agreement with previous reports [6]. The BJH pore size distribution confirms a multimodal distribution of pore sizes between 2 and 8 nm with averages at 3.7 and 5.3 nm confirmed by BJH adsorption and desorption, respectively. The identity of Pyr was confirmed via the (110), (101), (200), (111), and (210) hkl reflections [7], with bulk terminations defined by the (110), (101), and (100) faces [8–10]. The corresponding BJH distribution confirms the low porosity of the Pyr (Figure S1b), with an average porosity between 2-3 nm, and a pore volume 10% to that of Birn.

Ferrihydrite was confirmed by the presence of two broad reflections in the XRD pattern characteristic of 2-line Fhy corresponding to the (110) and (115) reflections [11,12]. The pore size distribution is centered at 1.5 nm. The second peak at 5.9 nm is a single data point observed only during the adsorption portion of the BJH analysis and likely represents an artifact. The total pore volume of the Fhy is comparable to Birn at > 0.1 cm^3 g^{-1}.

Goethite was confirmed by XRD to match previously published patterns of the pure mineral [13,14]. The narrow FWHM of the (110) hkl reflection indicates the predominance of this crystal face at the Gt surface, a characteristic of this Fe phase that has been reported previously [13,15–17]. The pore volume of Gt is approximately four times greater than that of Pyr, and half that of Fhy with pore diameters ranging from 2 and 4.5 nm depending on the BJH method used.

The purity of the Hm phase was confirmed at the resolution of XRD, with the ratio of the (012), (104), and (110) hkl reflections supporting a rhombohedral morphology [18]. This geometry has been described previously for Hm synthesized using low-temperature aqueous methods [19–22]. Studies of pH effects on Hm particle size resulting from $Fe(NO_3)_3$ hydrolysis have similarly shown rhombohedral particles of ~20 nm forming from conditions identical to those used in this study ($pH_{formation}$ = 2.7). [23] Although pore size was observed to be ~1 nm, the pore volume was comparable to that of Fhy. A possible explanation for this arises from the high surface area of our sample (61 m^2 g^{-1}), which is two times greater than has been reported previously using the same synthesis method; (Baltrusaitis et al., 2007; Schwertmann and Cornell, 2008) however, similar low-temperature hydrothermal methods have produced particles with comparable surface area [22]. Thus, a smaller particle size is expected and was observed with a small fraction of particles passing through the 0.22 μm membranes. Therefore, sedimentation was also used to separate the aqueous and solid phases on these samples prior to ICP-OES analysis.

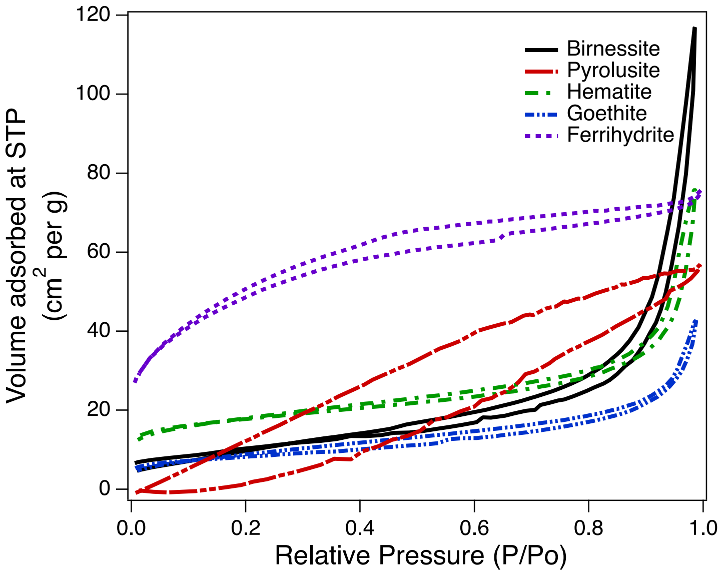

Figure S1a. Results of BET surface area analysis for each mineral species prior to reaction with V^{V}.

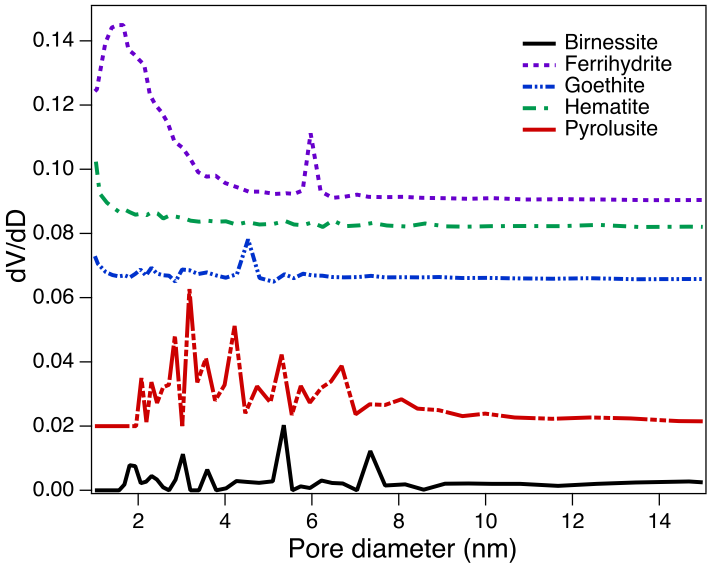

Figure S1b. Results of BJH porosity analysis for each mineral species prior to reaction with V^{V}.

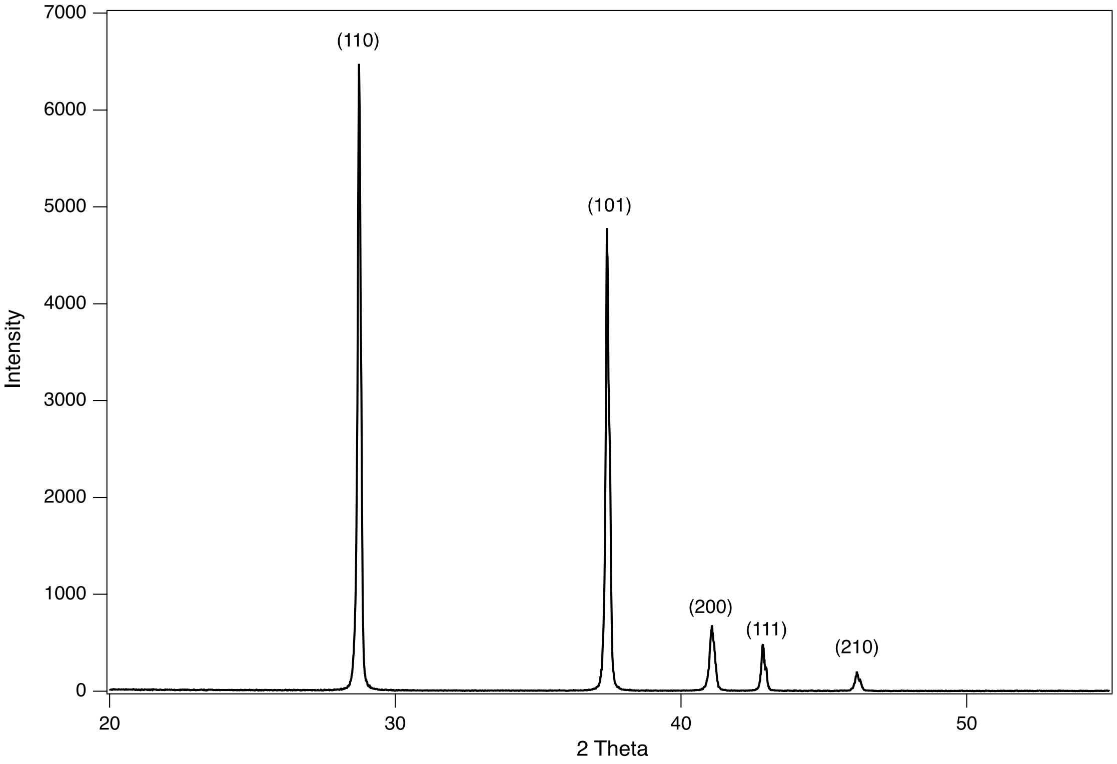

Figure S1c. XRD reflection for pyrolusite prior to reaction with V^{V}. Prominent (hkl) reflections are labelled.

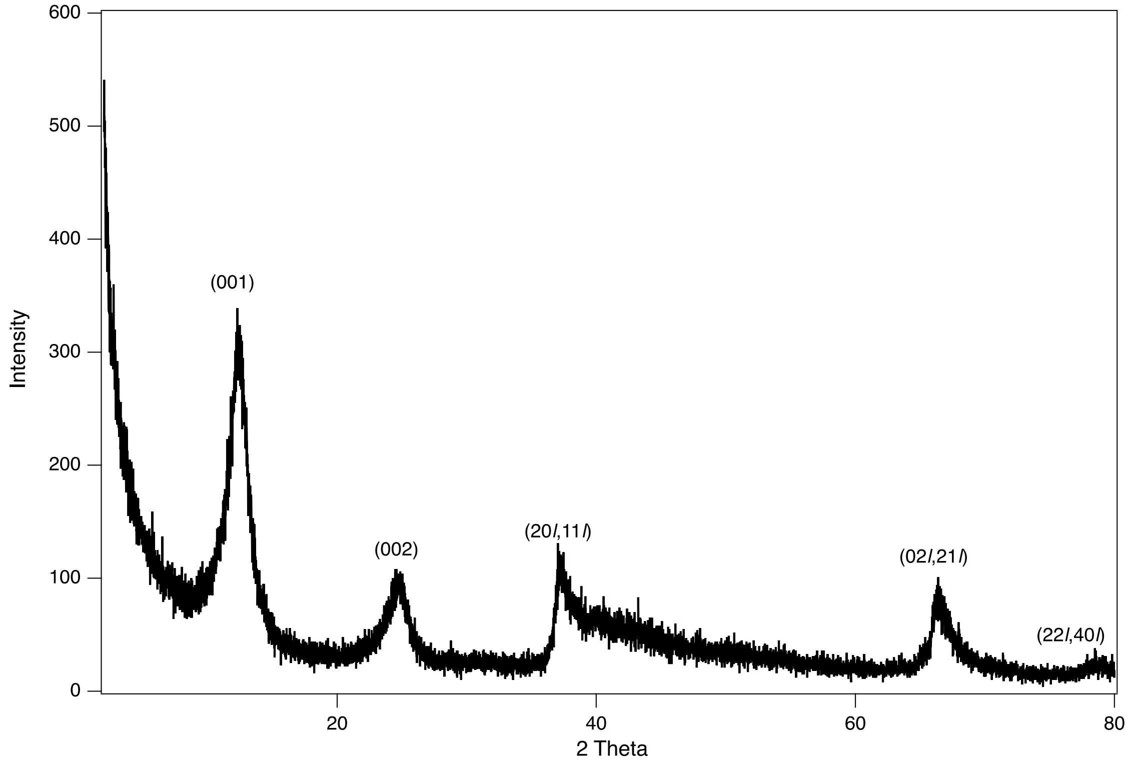

Figure S1d. XRD reflection for birnessite prior to reaction with V^V. Prominent (hkl) reflections are labelled.

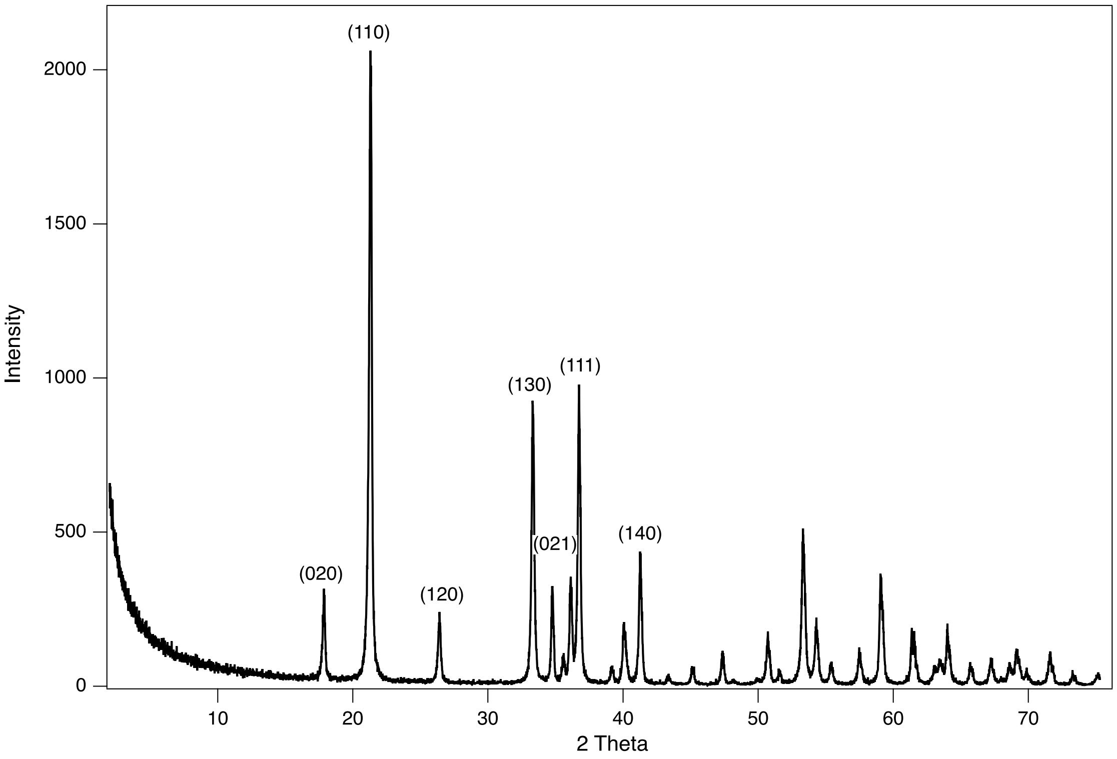

Figure S1e. XRD reflection for goethite prior to reaction with V^V. Prominent (hkl) reflections are labelled.

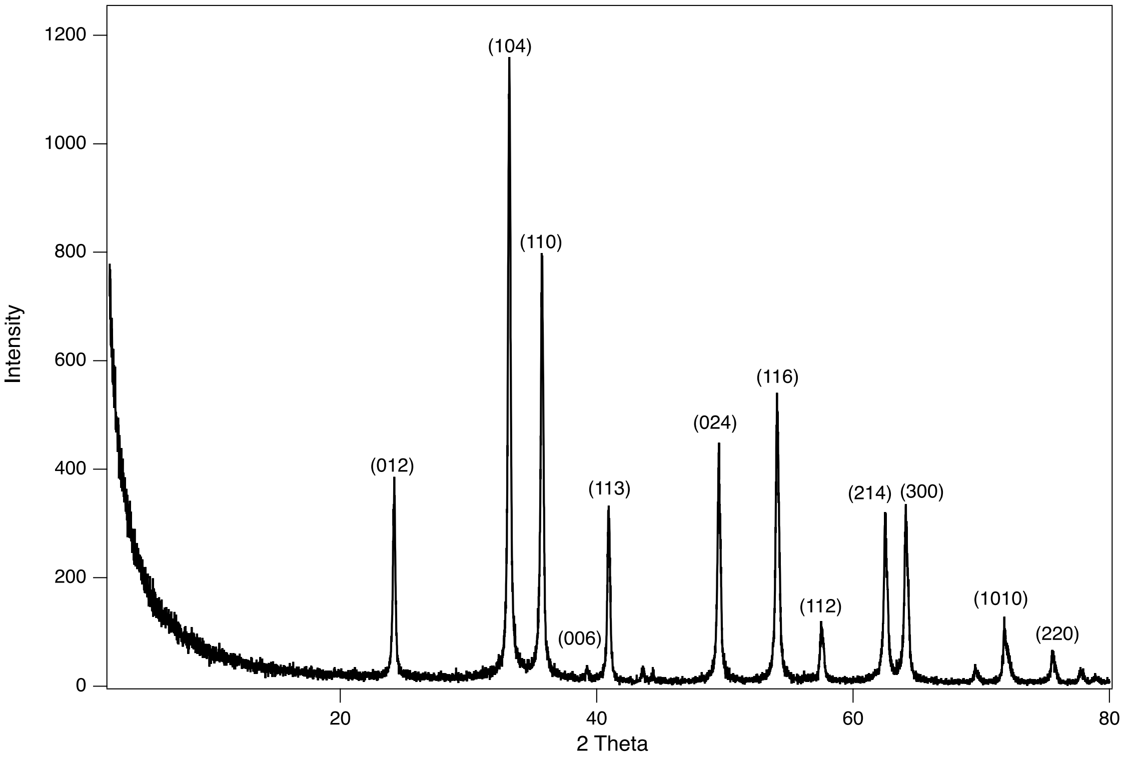

Figure S1f. XRD reflection for hematite prior to reaction with V^V. Prominent (hkl) reflections are labelled.

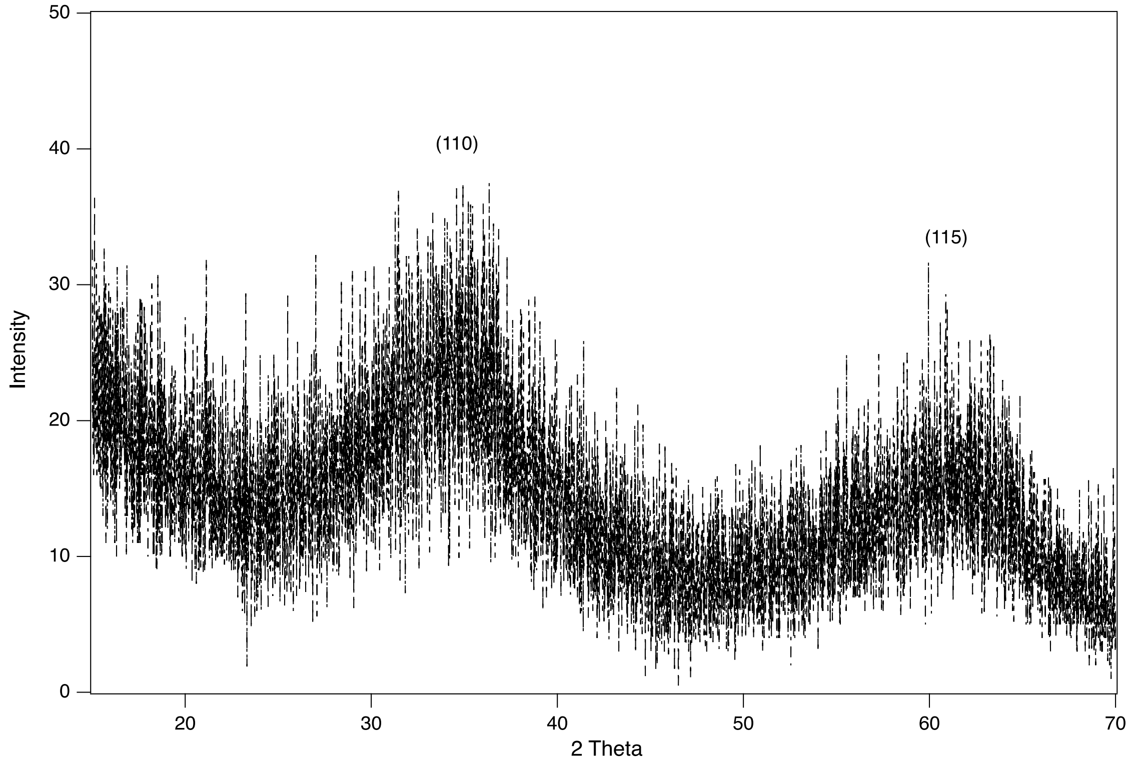

Figure S1g. XRD reflection for ferrihydrite prior to reaction with V^{V}. Prominent (hkl) reflections are labelled.

Section II. Transformed Isotherms

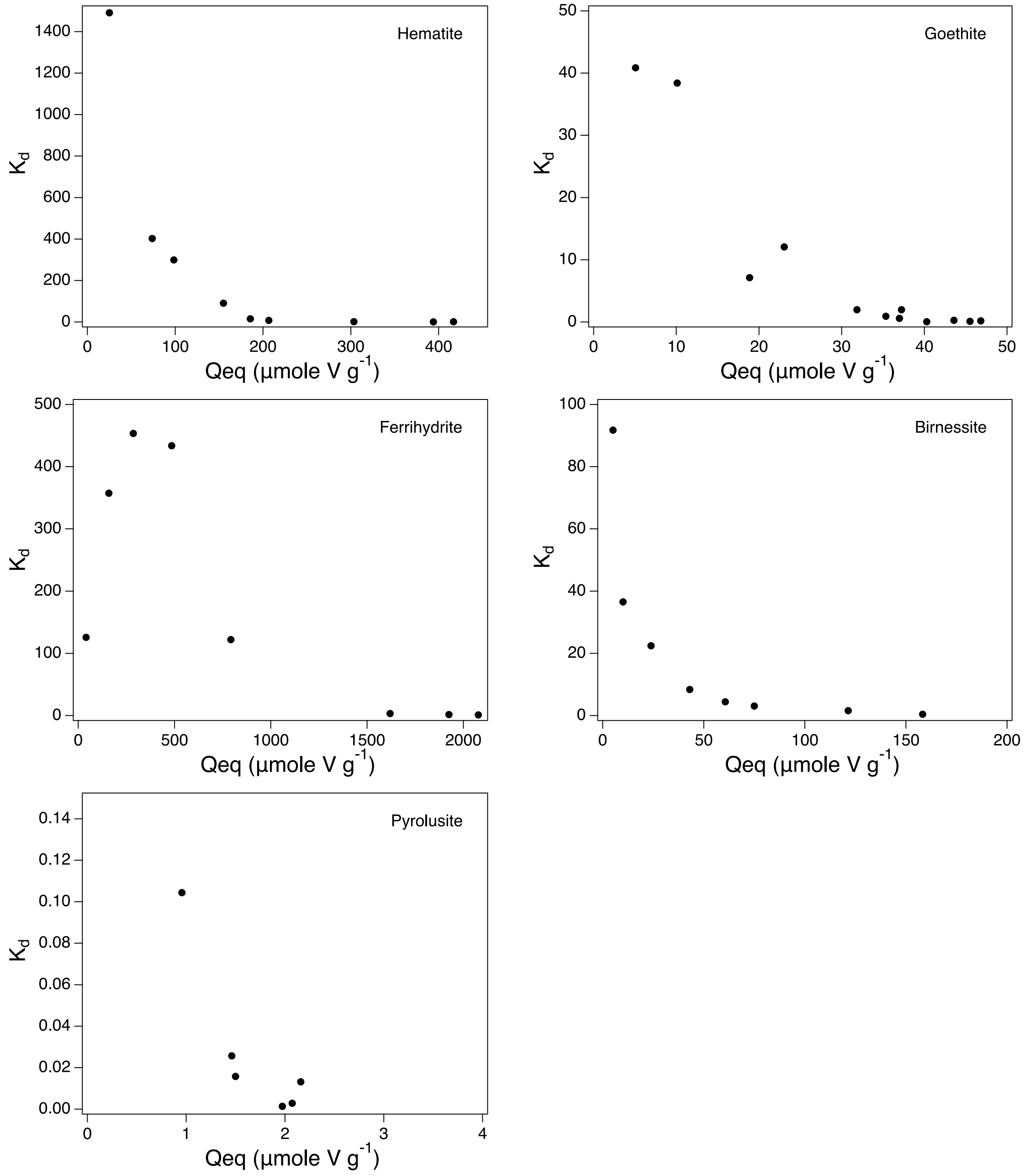

Figure S2. Scatchard plots for the adsorption of V^V onto each mineral species.

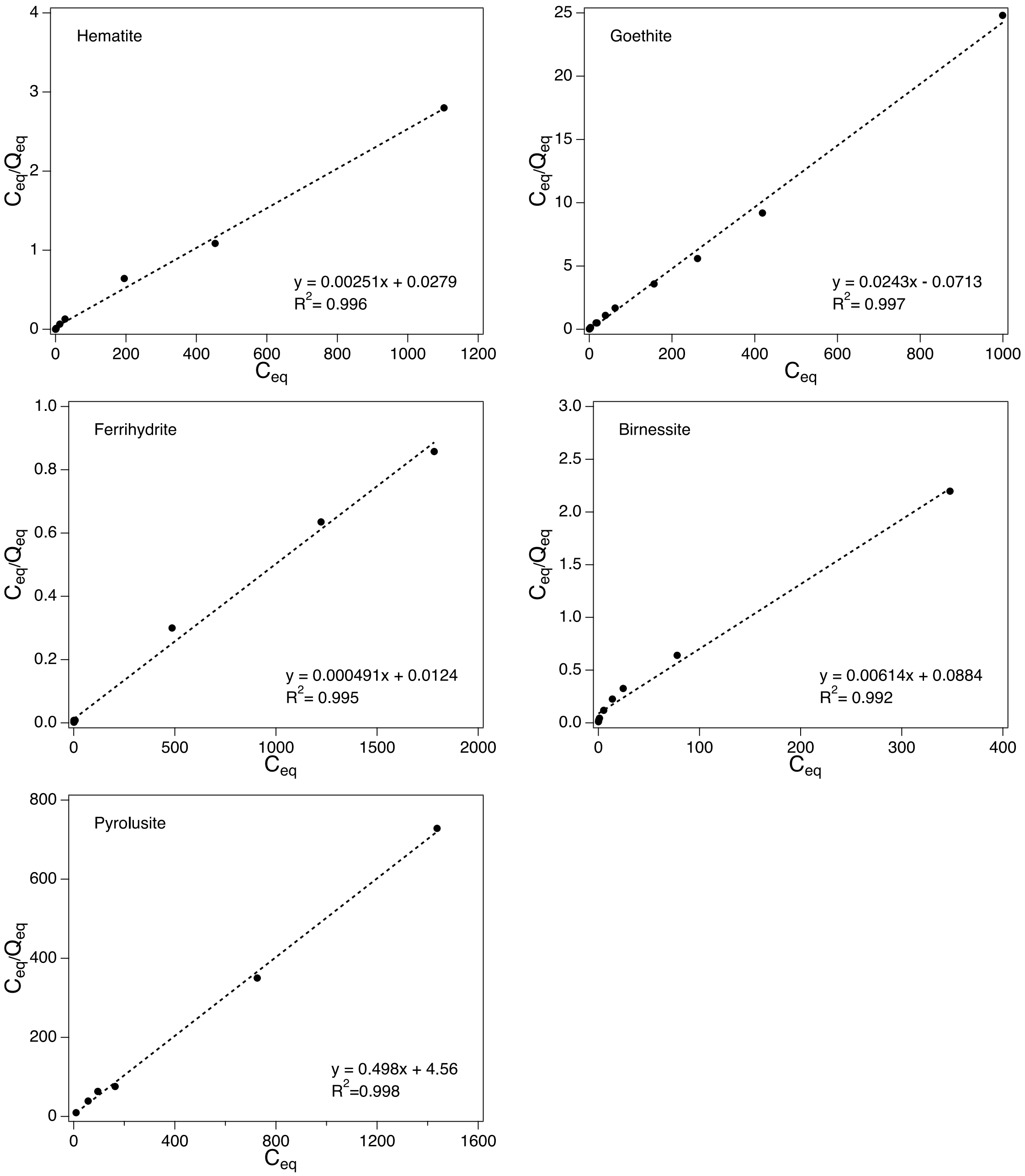

Figure S3. Linearized Langmuir plots of V^V onto each mineral species.

Section III. Aqueous V^V Speciation

Visual Minteq version 3.1 was used to calculate the V^V speciation for each equilibrium V^V_{aq} concentration obtained in the Langmuir isotherm experiments. Parameters for the calculation included 25 mM NaCl of background electrolyte, and the pH was fixed at 7 to account for the 10 mM of PIPES buffer.

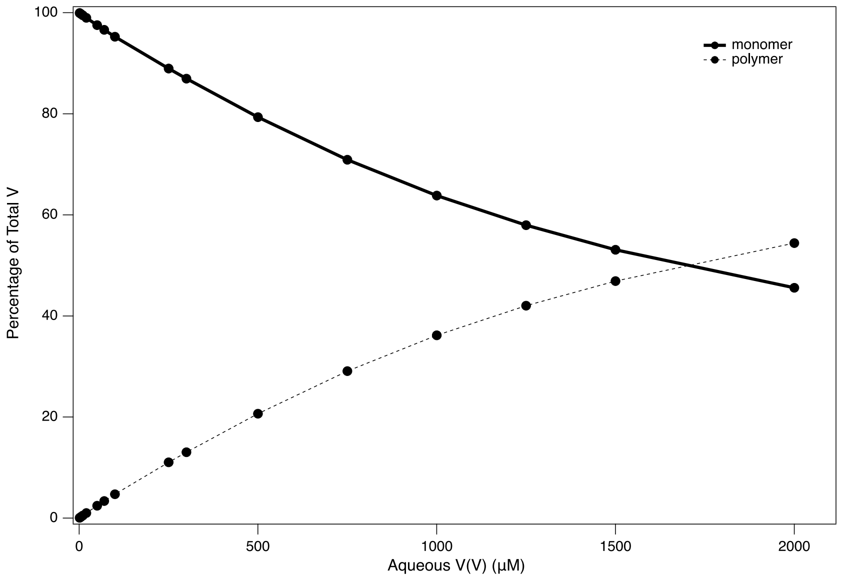

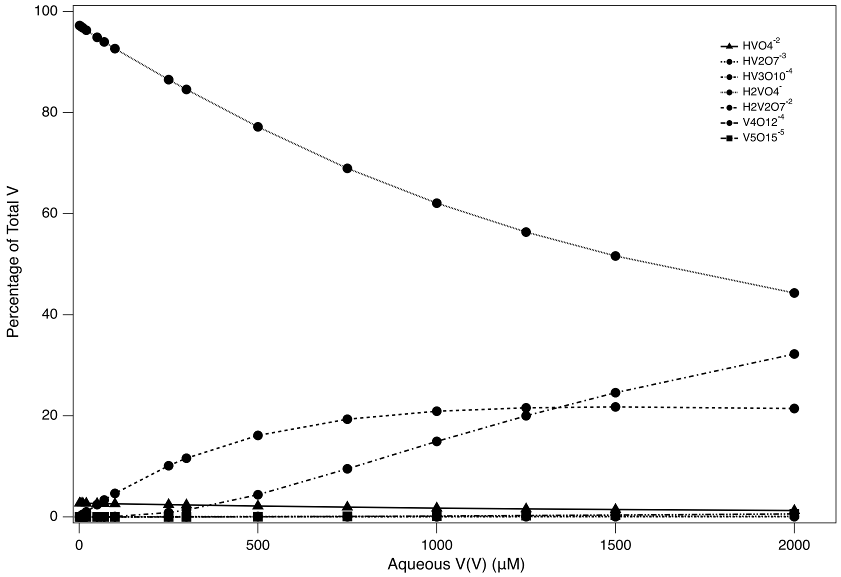

Figure S4. MINTEQ speciation results for V^{V} at pH 7 and 25 mM NaCl. A) monomeric vs polymeric fraction of V^{V}, where each category is a sum of the fractions of monomeric or polymeric species. B) The fraction of each V^{V} species at each concentration of total V^{V}.

Table S1. MINTEQ model results used to construct Figures 2.10 A and B.

Total V	Percent of Total V								
uM V(V)	HVO4-2	H2VO4-	H2V2O7-2	HV2O7-3	V4O12-4	V5O15-5	HV3O10-4	monomer	polymer
1	2.722	97.227	0.051	0	0	0	0	99.949	0.051
5	2.716	97.028	0.254	0	0	0	0	99.744	0.254
10	2.709	96.782	0.506	0	0	0	0	99.491	0.506
20	2.696	96.296	1.002	0	0	0	0	98.992	1.002
50	2.657	94.887	2.433	0.013	0	0	0	97.544	2.446
70	2.631	93.982	3.342	0.018	0.026	0	0	96.613	3.386
100	2.595	92.665	4.642	0.025	0.072	0	0	95.26	4.739
250	2.425	86.527	10.123	0.054	0.863	0	0	88.952	11.04
300	2.371	84.582	11.609	0.062	1.363	0	0	86.953	13.034
500	2.166	77.182	16.124	0.087	4.395	0.033	0.013	79.348	20.652
750	1.939	68.974	19.337	0.104	9.525	0.098	0.021	70.913	29.085
1000	1.748	62.076	20.913	0.113	14.934	0.185	0.027	63.824	36.172
1250	1.591	56.371	21.588	0.117	20.012	0.283	0.032	57.962	42.032
1500	1.461	51.637	21.773	0.119	24.583	0.385	0.036	53.098	46.896
2000	1.26	44.307	21.445	0.118	32.234	0.585	0.041	45.567	54.423

Section IV. Method for Nonlinear Least Squares Fitting

Microsoft excel was used to organize and refine the isotherm data to each of the isotherm models [25]. The standard deviation was calculated for each C_{eq} and Q_{eq} value, for each isotherm. For each isotherm model used, a modelled Q_{eq} value was determined according to equations 1 and 2, with approximate initial guesses placed for each of the dependent variables (q_{max}, K, K_L, etc). the residual was then calculated, taken as:

$$\text{residual} = \text{Q}_{\text{eq}_{\text{empirical}}} - \text{Q}_{\text{eq}_{\text{model}}}$$

The residuals were then squared to make all values positive. The squared residuals were then weighted by a factor w_i, where w_i is equal to the standard error of $\text{Q}_{\text{eq}_{\text{empirical}}}$, although other methods of weighting have been discussed [25]. The standard error of $\text{Q}_{\text{eq}_{\text{empirical}}}$ is defined as:

$$\text{SE} = \frac{\text{sandard deviation of Q}_{\text{eq}_{\text{empirical}}}}{\sqrt{Number\ of\ of\ replicates\ at\ each\ concentration}} = \frac{\text{sandard deviation of Q}_{\text{eq}_{\text{empirical}}}}{\sqrt{3}}$$

The weighted squared residuals were then summed (RSS), and the total number of data points in the isotherm (N) and total number of variables (m) used in the regression of $\text{Q}_{\text{eq}_{\text{model}}}$ on $\text{Q}_{\text{eq}_{\text{empirical}}}$ were counted. The root-mean squared error, also called the standard error [25] was then calculated according to:

$$\text{rmse} = \sqrt{\frac{RSS}{(N-m)}}$$

The rmse was then targeted as the parameter to be minimized by the optimization algorithm while adjusting the dependent variables. Additionally, the R^2 and 95% confidence intervals were calculated according to Brown (2001). However, the utility of an R^2 parameter in non-linear regression is low, primarily stemming from the requirement of an "mean" value of the empirically

determined independent variable. The representativeness of such a mean value towards its dataset is low for nonlinear data. Nonetheless, these values are reported for completeness.

Section V. Method for Birnessite Characterization

Determination of the number of coherently stacked layers in our birnessite sample were done using the collected XRD data, and results from Villalobos et al. (2006). Briefly, the crystallite sizes in the c* direction and for the ab plane were calculated using the Scherrer equation:

$$\tau = \frac{0.9\lambda}{\beta\cos(\theta)}$$

where τ is the crystallite size in nanometers, lambda is the wavelength of the x-rays used (Cu K_{α} = 0.154059 nm), β is the full width at half-maximum intensity of the XRD reflection in radians, and θ is the angle at which the reflection occurs, in radians. To calculate the distance along the c* direction, the (001) reflection was used, yielding a distance of 6.4 nm. To translate this into the number of turbostratically stacked layers, data from Villalobos et al. was used. Linear regression was applied to the reported values for length along the c* axis, and the corresponding number of layers. (crystallite size along the c* direction in nm = independent variable, number of layers = dependent variable)

Table S2. Parameters used to calculate the number of stacked birnessite layers

c* nm	layers
1.9	2.6
2	2.8
4.2	5.8

Data collected by Villalobos et al. (2006) used to determine the number of coherently stacked layers of birnessite.

Regression results: number of layers = 1.379*(c* nm) + 0.0108

Thus, an average of 8.8 layers were determined for our birnessite, having an average c* extent of 6.4 nm.

X-ray absorption spectroscopy on birnessite before and after incubation with the PIPES-buffered Na_3VO_4 was also done to monitor any PIPES-induced changes in the birnessite structure, as Good's buffers have been shown to act as reductants towards Mn(IV) oxides [26]. Spectra were collected at SSRL on beamline 4-3 using an Si(111) crystal monochrometer with an orientation of φ= 90. Substantial crystal glitches, which begin to appear in the χ(k) data at 11.8 $Å^{-1}$ limited the EXAFS range to k = 12 $Å^{-1}$. The spectra for unreacted birnessite was collected at beamline 7-3 with an Si(220) crystal set oriented to φ = 90. All data were collected in fluorescence mode with a PIPS detector, chromium filter and Soller slits at room temperature with an in-line Mn foil with the maximum of the first derivative set to 6539 eV. After incubation with the buffered vanadate solution, all samples displayed an edge shift when the normalized intensity was equal to 0.5 ($E_{1/2}$) to lower energies of approximately 1 eV. The $E_{1/2}$ value Birnessite was 6551.5 eV, which shifted to 6550 eV in the 75 μM V(V) sample, 6550.3 in the 25 μM V(V) sample and 6550.9 eV in the 500 μM V(V) sample (Figure S5a). Despite this shift, the EXAFS data show that, rather than forming a secondary Mn(III) hydroxide phase, the Mn(IV) reduced by the PIPES adsorbed to surface vacancy sites in the birnessite in a triple-corner-sharing manner. This is seen in the increase in the shoulder feature at 3.5 Å in the Fourier-transformed EXAFS plot, as well as the formation of a shoulder at 6.5 $Å^{-1}$ in the k^3-weighted EXAFS (Figure S5 b,c) [3]. In a previous study, we demonstrated that Mn(II) generated from the reduction of structural Mn(IV) in birnessite and V(V) do not compete for the same adsorption sites, with Mn(II) adsorbing primarily to cap surface vacancies and V(V) predominately adsorbing to edge sites [27].

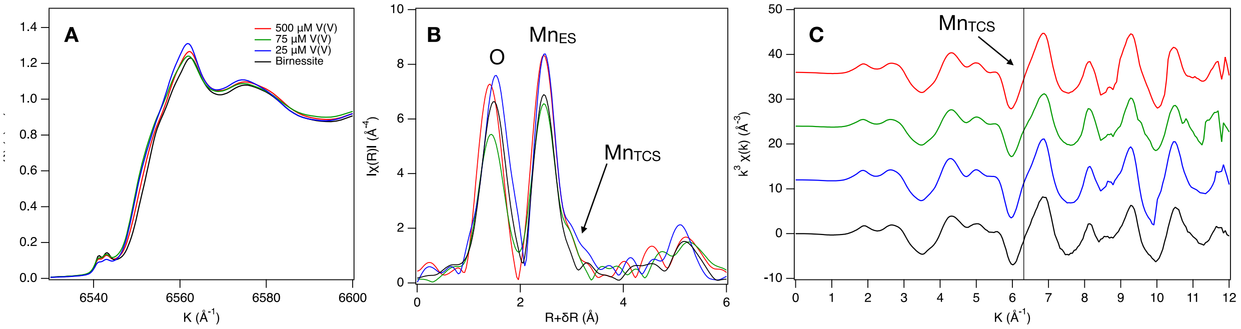

Figure S5. Mn K-edge XAS of before and after incubation with the buffered V(V) solution at 25 μM, 75 μM and 500 μM Na_3VO_4. (A) XANES showing a 1 eV shift to lower energy after incubation, indicating the reduction of structural Mn(IV) to Mn(II) and Mn(III). (B) Fourier-transformed EXAFS showing the location of the octahedrally coordinating O, edge-sharing (es) Mn and triple-corner-sharing Mn. (C) k^3-weighted EXAFS with an indicator line at 6.5 $Å^{-1}$ where the presence of Mn_{TCS} adsorbed to vacancy sites results in the formation of a weak shoulder feature.

Section VII. Mineral Synthesis

Birnessite

63 g of $KMnO_2$ (Fisher) was dissolved into 1 L of ultrapure water (EMD Millipore) and heated to 90°C. Sixty-six ml of concentrated trace metal grade HCl was slowly added over the course of ten minutes. The mixture was then maintained at 90°C for 10 minutes before cooling to 21°C over the course of 30 minutes. Manganese oxides in the solution were collected using vacuum filtration. The Mn oxides were repeatedly rinsed with ultrapure water to remove excess $KMnO_4$ prior to air-drying.

Hematite

A 2 mM solution of trace metal grade HNO_3 was prepared, capped and brought to 98 °C in an oven. When the temperature was reached, 17 g of $Fe(NO_3)•9H_2O$ was added with stirring. The solution was then returned to the oven and held at 95-100 °C for a week. Solids were separated by centrifugation, and rinsed with ultrapure water before air-drying.

2-line Ferrihydrite

43.7 g of $Fe(NO_3)•9H_2O$ was dissolved in 500 ml of ultrapure water. 95 ml of 2 molar KOH was then added, with an additional 10 ml added dropwise. The resulting precipitate was then filtered, rinsed with ultrapure water and air dried.

Section VIII. References

(1) Lopano, C. L.; Heaney, P. J.; Post, J. E.; Hanson, J.; Komarneni, S. Time-Resolved Structural Analysis of K- and Ba-Exchange Reactions with Synthetic Na-Birnessite Using Synchrotron X-Ray Diffraction. *Am. Mineral.* **2007**, *92* (2–3), 380–387. https://doi.org/10.2138/am.2007.2242.

(2) Grangeon, S.; Lanson, B.; Lanson, M.; Manceau, A. Crystal Structure of Ni-Sorbed Synthetic Vernadite: A Powder X-Ray Diffraction Study. *Mineral. Mag.* **2008**, *72* (6), 1279–1291. https://doi.org/10.1180/minmag.2008.072.6.1279.

(3) Villalobos, M.; Lanson, B.; Manceau, A.; Toner, B.; Sposito, G. Structural Model for the Biogenic Mn Oxide Produced by Pseudomonas Putida. *Am. Mineral.* **2006**, *91* (4), 489–502. https://doi.org/10.2138/am.2006.1925.

(4) Drits, V. A.; Lanson, B.; Gaillot, A.-C. Birnessite Polytype Systematics and Identification by Powder X-Ray Diffraction. *Am. Mineral.* **2007**, *92* (5–6), 771–788. https://doi.org/10.2138/am.2007.2207.

(5) Lanson, B.; Marcus, M. A.; Fakra, S.; Panfili, F.; Geoffroy, N.; Manceau, A. Formation of Zn–Ca Phyllomanganate Nanoparticles in Grass Roots. *Geochim. Cosmochim. Acta* **2008**, *72* (10), 2478–2490. https://doi.org/10.1016/j.gca.2008.02.022.

(6) Marafatto, F. F.; Lanson, B.; Peña, J. Crystal Growth and Aggregation in Suspensions of δ-MnO2 Nanoparticles: Implications for Surface Reactivity. *Environ. Sci. Nano* **2018**, *5* (2), 497–508. https://doi.org/10.1039/C7EN00817A.

(7) Wyckoff, R. Crystal Structures. **1963**.

(8) Schaefer, M. V.; Handler, R. M.; Scherer, M. M. Fe(II) Reduction of Pyrolusite (β-MnO2) and Secondary Mineral Evolution. *Geochem. Trans.* **2017**, *18*. https://doi.org/10.1186/s12932-017-0045-0.

(9) Yamada, N.; Ohmasa, M.; Horiuchi, S. Textures in Natural Pyrolusite, β-MnO2, Examined by 1 MV HRTEM. *Acta Crystallogr. B* **1986**, *42* (1), 58–61. https://doi.org/10.1107/S0108768186098579.

(10) Maphanga, R. R.; Parker, S. C.; Ngoepe, P. E. Atomistic Simulation of the Surface Structure of Electrolytic Manganese Dioxide. *Surf. Sci.* **2009**, *603* (21), 3184–3190. https://doi.org/10.1016/j.susc.2009.07.038.

(11) Wooyong Um, M. M. S. Adsorption Mechanisms and Transport Behavior between Selenate and Selenite on Different Sorbents. *Int. J. Waste Resour.* **2014**, *04* (02). https://doi.org/10.4172/2252-5211.1000144.

(12) Janney, D. E.; Cowley, J. M.; Buseck, P. R. Structure of Synthetic 2-Line Ferrihydrite by Electron Nanodiffraction. **2000**, 8.

(13) Gualtieri, A. F.; Venturelli, P. In Situ Study of the Goethite-Hematite Phase Transformation by Real Time Synchrotron Powder Diffraction. *Am. Mineral.* **1999**, *84* (5–6), 895–904. https://doi.org/10.2138/am-1999-5-624.

(14) Notini, L.; Latta, D. E.; Neumann, A.; Pearce, C. I.; Sassi, M.; N'Diaye, A. T.; Rosso, K. M.; Scherer, M. M. The Role of Defects in Fe(II)–Goethite Electron Transfer. *Environ. Sci. Technol.* **2018**, *52* (5), 2751–2759. https://doi.org/10.1021/acs.est.7b05772.

(15) Burleson, D. J.; Penn, R. L. Two-Step Growth of Goethite from Ferrihydrite. *Langmuir* **2006**, *22* (1), 402–409. https://doi.org/10.1021/la051883g.

(16) Ostergren, J. D.; Trainor, T. P.; Bargar, J. R.; Brown, G. E.; Parks, G. A. Inorganic Ligand Effects on Pb(II) Sorption to Goethite (α-FeOOH): I. Carbonate. *J. Colloid Interface Sci.* **2000**, *225* (2), 466–482. https://doi.org/10.1006/jcis.1999.6701.

(17) Weidler, P. G.; Hug, S. J.; Wetche, T. P.; Hiemstra, T. Determination of Growth Rates of (100) and (110) Faces of Synthetic Goethite by Scanning Force Microscopy. *Geochim. Cosmochim. Acta* **1998**, *62* (21), 3407–3412. https://doi.org/10.1016/S0016-7037(98)00251-8.

(18) Sugimoto, T.; Muramatsu, A.; Sakata, K.; Shindo, D. Characterization of Hematite Particles of Different Shapes. *J. Colloid Interface Sci.* **1993**, *158* (2), 420–428. https://doi.org/10.1006/jcis.1993.1274.

(19) Baltrusaitis, J.; M. Cwiertny, D.; H. Grassian, V. Adsorption of Sulfur Dioxide on Hematite and Goethite Particle Surfaces. *Phys. Chem. Chem. Phys.* **2007**, *9* (41), 5542–5554. https://doi.org/10.1039/B709167B.

(20) Jang, J.-H.; Dempsey, B. A.; Burgos, W. D. Solubility of Hematite Revisited: Effects of Hydration. *Environ. Sci. Technol.* **2007**, *41* (21), 7303–7308. https://doi.org/10.1021/es070535t.

(21) Schwertmann, U.; Cornell, R. *Iron Oxides in the Laboratory: Preparation and Characterization*; Wiley & Sons, 2008.

(22) Frierdich, A. J.; Helgeson, M.; Liu, C.; Wang, C.; Rosso, K. M.; Scherer, M. M. Iron Atom Exchange between Hematite and Aqueous Fe(II). *Environ. Sci. Technol.* **2015**, *49* (14), 8479–8486. https://doi.org/10.1021/acs.est.5b01276.

(23) Jolivet, J.-P.; Chanéac, C.; Tronc, E. Iron Oxide Chemistry. From Molecular Clusters to Extended Solid Networks. *Chem. Commun.* **2004**, *0* (5), 481–483. https://doi.org/10.1039/B304532N.

(24) Schwertmann, U.; Cornell, R. *Iron Oxides in the Laboratory: Preparation and Characterization*; Wiley.

(25) Brown, A. M. A Step-by-Step Guide to Non-Linear Regression Analysis of Experimental Data Using a Microsoft Excel Spreadsheet. *Comput. Methods Programs Biomed.* **2001**, *65* (3), 191–200. https://doi.org/10.1016/S0169-2607(00)00124-3.

(26) Simanova, A. A.; Kwon, K. D.; Bone, S. E.; Bargar, J. R.; Refson, K.; Sposito, G.; Peña, J. Probing the Sorption Reactivity of the Edge Surfaces in Birnessite Nanoparticles Using Nickel(II). *Geochim. Cosmochim. Acta* **2015**, *164*, 191–204. https://doi.org/10.1016/j.gca.2015.04.050.

(27) Abernathy, M. J.; Schaefer, M. V.; Vessey, C. J.; Liu, H.; Ying, S. C. Oxidation of V(IV) by Birnessite: Kinetics and Surface Complexation. *Environ. Sci. Technol.* **2021**. https://doi.org/10.1021/acs.est.1c02464.
